# Sociodemographic and work-related differences in teachers’ attitude towards and perceived stress from emergency remote teaching during the COVID-19 pandemic

**DOI:** 10.1038/s41598-023-39824-w

**Published:** 2023-08-10

**Authors:** Kristin Kalo, Clemens Koestner, Theresa Dicks, Viktoria Eggert, Till Beutel, Carolina Zähme, Stephan Letzel, Pavel Dietz

**Affiliations:** 1 Department of Sports Medicine, Disease Prevention and Rehabilitation, Johannes Gutenberg University Mainz, 55128 Mainz, Germany; 2 Institute of Occupational, Social and Environmental Medicine, University Medical Center of the University of Mainz, Mainz, Germany; 3 Institute for Teachers’ Health, University Medical Center of the Johannes Gutenberg University of Mainz, Mainz, Germany

**Keywords:** Psychology, Occupational health

## Abstract

The aim was to investigate the attitude towards and perceived stress from emergency remote teaching (ERT) among teachers during the COVID-19 pandemic. A Germany-wide online survey was conducted among teachers from all school types in March 2021. Data from 31,089 teachers entered analysis. ANOVAs or Welch’s t-tests with post-hoc analyses were performed to determine sociodemographic and work-related group differences in teachers’ attitude towards and perceived stress from ERT. The mean attitude towards ERT was 3.47 (± .84) out of 5 and the mean perceived stress was 5.03 (± .62) out of 6. Regarding the attitude towards ERT, we revealed significant differences for gender, age groups, number of children, occupational group, school management membership, and employment status (*p* < .05). Regarding perceived stress, significant differences were obtained for gender, age groups, and employment status (*p* < .05). A more positive attitude towards ERT seems to be associated with lower stress levels. Being female, a higher age, a higher number of children living in the own household as well as working full-time might hinder an effective implementation of remote teaching in school settings in Germany. Policy-makers and schools should think of strategies to improve the attitude towards and decrease perceived stress from remote teaching. This could include subgroup-specific training on the use of digital media, adapted to the work environment.

## Introduction

In reaction to the COVID-19 pandemic, all schools in Germany were closed in March 2020. Even though there was a partial re-opening of schools 2 months later, this was accompanied by severe restrictions and changes in the living and working environment of teachers^1^. Consequently, in the period from March 2020 to March 2022 teachers had to partly or even completely switch from face-to-face classes to remote teaching, a distance learning methodology using online platforms and multimedia techniques^2^. Working from home, teachers became increasingly dependent on digital tools for both teaching and communicating with colleagues, parents, and students. The pandemic has shown that neither schools nor their teachers were well prepared to manage the multiple challenges that came along with this change, e.g., the dependency on digital tools for both teaching and communication processes, the competent and conducive use of digital media for learning purposes, and maintaining the relationship with the students^3,4^. Therefore, remote teaching is considered not only an opportunity, but an increased burden to teachers as well^5^.

It seems to be advantageous that the transition to mostly remote teaching occurred in the midst of an ongoing digitalization process of the German education system^6^. In this context, digitalization refers to the sociotechnical processes associated with the use of digital technologies, which impact social and institutional contexts that require and increasingly rely on digital technologies^7^. The Standing Conference of the Ministers of Education and Cultural Affairs of the Federal States (2016) created the strategy “Education in the Digital World” (*Bildung in der digitalen Welt)* to further expand teaching and learning with the help of digital media (e.g., technical equipment like laptops or tablets, online learning platforms like Moodle or Microsoft Teams, electronic learning materials like eBooks, videos and podcasts) and in digital learning environments (e.g., training courses for relevant pedagogical and technical skills) even years before the onset of the pandemic^6,8^. In addition, the Federal Ministry of Education and Research provided a budget of EUR 5 billion to equip schools with appropriate information and communication technology^6^. However, despite these efforts to digitalize the German education system, recent research showed that other European countries already use digital infrastructures and web-based learning tools to a greater extent than Germany^6,9,10^. Eventually, the COVID-19 pandemic just re-emphasizes the relevance of a digital transformation of German schools as part of an increasingly digitalized society. Now, more than ever, there is a need to develop strategies for integrating information and communication technologies in German schools. In such a digitalization process, teachers play an essential role as they have to integrate those technologies into their work environment and their classroom as well as impart them to their pupils^1,4^. Therefore, it is important to know about the factors that support or impede teachers in successfully implementing remote teaching. Because the remote teaching was not planned and designed as an online class from the beginning and was used as an emergency format during the COVID-19 pandemic, it can be seen as a temporary shift of instructional delivery to an alternate delivery mode due to crisis circumstances^11^. Hereafter, we will refer to as emergency remote teaching (ERT), when the term is directly related to the COVID-19 pandemic and as remote teaching when considered independent of the pandemic.

Previous studies investigated determinants for the implementation of remote teaching in different educational settings. These studies revealed that teachers’ beliefs in effective learning through digital media and their attitude towards remote teaching were strong predictors for a successful integration in class^9,12–16^. Teachers’ knowledge and (technical) skills as well as their self-efficacy in remote teaching were cited as further influencing factors and were closely related to their beliefs and attitudes^3,12^. Košir et al.^13^ showed that teachers who reported higher self-efficacy in using information and communication technology had a more positive attitude towards ERT during pandemic.

While researchers substantially agree that teachers’ attitude support or hinder the implementation of ERT, the body of research on teachers’ actual attitude is less consistent^13,17–19^. The reason might be, that the exact nature of attitudes is unclear^12^. Moreover, Košir et al.^13^ revealed the attitude towards ERT as a significant predictor for the experienced stress level of teachers. High stress levels, in turn, hinder teachers’ willingness and ability to implement innovative practices like remote teaching^20^. The teaching profession was already characterized by high levels of stress even before the COVID-19 pandemic^21^. Causes for this included classroom management, political changes affecting the curriculum or work-privacy-conflicts. During the COVID-19 pandemic, many of these difficulties seem to come together all at once, along with the stress caused by the sudden switch from traditional teaching to ERT^22^. Nevertheless, research investigating stress levels of teachers during the COVID-19 pandemic show inconsistent results^21,23–25^. In order to target the attitude towards ERT and counteract a pandemic-related increase in stress, it is important to know characteristics and factors influencing these two variables (i.e., attitude towards ERT and stress from ERT).

Teachers particularly in Germany reported rather a positive attitude towards ERT^10,12^, but also medium to high levels of stress^3,10,26^. In addition, Drossel et al.^27^ showed that older and female teachers in Germany had greater concerns about the use of digital media than younger or male colleagues. Moreover, teachers serving in the highest track of secondary school seemed to feel more prepared for remote teaching than those teaching in lower tracks of secondary school or in primary school^27^. However, we still do not know much about the sociodemographic and work-related factors that account for the differences in teachers’ attitude towards and their perceived stress from remote teaching, especially during the pandemic (ERT).

In order to address this knowledge gap, the present study aimed a) to investigate the attitude towards and perceived stress from suddenly switching to ERT among German teachers during the COVID-19 pandemic in order to b) assess group differences with regard to sociodemographic (i.e., gender, age, and children in household) and work-related (i.e., school type, occupational group, being part of the school management, and full-time employment) characteristics. This may enable us to identify characteristics in teachers that indicate increased difficulties with ERT to develop further education and support with remote teaching for specific groups of teachers.

Based on our literature search, we expect that female and older teachers^14^, as well as teachers with many children in their own household, will have a more negative attitude towards and higher perceived stress levels from ERT than male and younger teachers, or teachers with no or just one child in their household. In addition, we assume a more negative attitude towards and higher perceived stress levels from ERT in teachers working at lower educational tracks or with younger pupils in comparison to teachers working at higher educational tracks or with older pupils^27^. We hypothesize that teachers’ attitude and perceived pandemic-related stress levels differ by occupational group, membership of school management and working hours at school.

## Methods

### Study design and survey procedure

In March 2021, a cross-sectional, nationwide online survey was conducted among teachers in Germany as part of the ‘SARS-CoV-2 occupational and infection control measures in schools’ project^26^. All teachers who were employed part-time or full-time at a school in Germany at that time were eligible to participate in the survey. We have included all types of schools that currently exist in Germany. Participants were recruited with the support of governmental (Ministry of Education in Rhineland-Palatinate) and non-governmental institutions (Education and Science Workers’ Union), teacher-related societies (German Teachers Association), and projects associated with education (Monitor Lehrerbildung). A non-monetary incentive (EUR 2000.00 donation to the German Children’s Fund) was offered to foster the willingness to participate. The survey was online for 31 days. A total number of 39,359 teachers participated in the survey.

Before March 2021, we conducted a pilot phase to test the suitability of the questionnaire. At first, we asked (a) colleagues and afterwards (b) selected teachers to answer the questionnaire. In (a), we have determined the response time for the entire questionnaire, which was about 35 min. In (b), we discussed the questionnaire in detail with selected teachers in order to identify and adjust language or content-related problems.

The survey was approved by the ethics committee of the Medical Association of Rhineland-Palatinate and conducted in accordance with the standards set by the declaration of Helsinki (application-number: 2020-15531). All participants provided informed consent digitally.

### Measures

The survey was conducted using the web-based software LimeSurvey (LimeSurvey GmbH, Hamburg) and contained a total of 353 questionnaire items, which were arranged under following categories: (1) sociodemographic and work-related information, (2) identification of pandemic-specific burdens and challenges for teachers, (3) implementation, communication, and compliance with hygiene policies, (4) impact of school operations during the COVID-19 pandemic on teachers, and (5) collection of good practice examples^26^. The present study focused on category 2 (pandemic-specific burdens and challenges) and dealt with self-designed questions regarding the attitude of teachers towards and their perceived stress from ERT. The survey was administered in German (see Supplementary Information). The following questions were translated into English based on the back-translation method of Tyupa^28^.

#### Attitude towards emergency remote teaching

Teachers’ attitudes towards ERT were measured by rating their agreement to the following statements on a five-point Likert scale as ‘strongly disagree’, ‘somewhat disagree’, ‘neither agree nor disagree, ‘somewhat agree’, ‘strongly agree’. The translated statements were: (1) “I consider the use of remote teaching formats overall as positive.”, (2) “The use of remote teaching formats had a positive impact on the performance level of my students.”, (3) “I consider the use of remote formats as an opportunity.”. Subsequently, a sum scale was calculated (3 items, Cronbach’s α = .79).

#### Perceived stress from emergency remote teaching

Teachers were asked what changes they observed in school operations because of the COVID-19 pandemic. The following translated questions or statements could be answered with “yes” or “no”: (1) “Did you (at times) switch from face-to-face to remote teaching during the COVID-19 pandemic?”, (2) “Remote teaching was complicated by technical problems (e.g., disconnections, software errors, operating problems).”, (3) “Remote teaching was complicated by insufficient technical equipment (e.g., non-existent or outdated equipment).“, (4) “Remote teaching entailed that you often felt overwhelmed by your tasks.” If teachers answered the question or statement with “yes”, a second question followed asking to what extent they felt stressed by this change. Perceived stress from ERT was rated on a six-point Likert scale as ‘not at all’, ‘to a very small extent’, ‘to a small extent’, ‘to some extent’, ‘to a large extent’, ‘to a very large extent’. Subsequently, a sum scale was calculated (4 items, Cronbach’s α = .66).

#### Sociodemographic and work-related characteristics

With regard to sociodemographic characteristics, age, gender and number of children living in the household were assessed. Moreover, we acquired work-related characteristics such as type of school (described below), occupational group (teacher, teaching aid, candidate), whether teachers were members of the school management team (yes, no), and employment status (full-time, part-time).

The German educational system is structured differently compared to other countries. Our classification of school types was based on the official classification of the federal government^29^. After completing their primary school (grades 1–4) and depending on their performance in primary school, children (without special educational needs) can attend one of four types of secondary schools in Germany: (1) secondary general school (low educational track, grades 5–9/10), (2) secondary school (middle educational track, grades 5–10), (3) secondary academic school (high educational track, grades 5–12/13), (4) comprehensive school (combination of all three educational tracks, grades 5–12/13). Germany has special needs schools for nine different categories of disabilities (e.g., learning disability, physical disability, intellectual disability, and chronical illness)^30^. The German vocational education system combines firm-based training programs with a school-based component (one to 2 days per week of vocational school), in which apprentices acquire upper secondary general education in core subjects (like math and German) and theoretical knowledge in their training occupation (dual training system)^31^. Finally, we surveyed teachers from all school types (primary school, secondary general school, secondary school, secondary academic school, comprehensive school, special needs school, vocational school) and others, if the teachers could not be clearly assigned to one of the mentioned types (e.g., postgraduate vocational schools).

### Data processing and statistics

Our a priori sample size calculation was based on standard assumptions and size of the actual population of teachers in Germany in 2020/2021 (N = approx. 800,000). We considered a two-sided alpha-error (α) level of 1% and a 95% confidence interval. Accordingly, we needed data from at least 9491 participants.

Data cleaning was carried out to exclude cases that had dropped out at the beginning of the survey or had only answered the sociodemographic questions. Likewise, implausible values (e.g., stated age out of the working age range: below 18 years or above 67 years) were marked as missing, and duplicates were removed from the data set.

Participants’ age was categorized into four age quartiles using Tukey’s Hinges: The first quartile ranges from 18 to 37 years, the second quartile from 38 to 46 years, the third quartile from 47 to 54 years, and the fourth quartile from 55 to 67 years.

Before inference statistical analyses were conducted, data distribution and variance homogeneity were tested. A correlation between attitude towards and stress from ERT was carried out. To determine potential group differences in teachers’ attitude towards and perceived stress from ERT regarding sociodemographic and work-related characteristics, we calculated analyses of variance (ANOVAs) or Welch’s t-tests, respectively. Post-hoc analyses using Bonferroni (ANOVAs) or Games-Howell (Welch’s t-tests) correction were performed. For the estimates of effect sizes the eta squared (η^2^) was used and interpreted according to Cohen^32^: .01 (small effect), .06 (medium effect) and .14 (large effect). All analyses were performed using SPSS version 27 (IBM Corporation, New York, NY, USA); an alpha-error of 5% was considered as a relevant cut-off significance value.

## Results

A total of 39,359 teachers from German schools participated in the survey, and data of 31,089 teachers remained after data cleaning for further analyses. Overall, 77.52% (n = 24,099) participants were female, 22.04% (n = 6851) were male and .45% (n = 139) identified as diverse. The age ranged from 18 to 67 years (M = 45.8 ± 10.5). The number of children in the household ranged from 0 to 9 and is subdivided as follows: 56.60% (n = 16,468) participants had no children, 17.47% (n = 5082) stated to have one child, 20.07% (n = 5839) had two children and 5.87% (n = 1707) had three or more children in their household. The number of participating teachers could be allocated to school types as follows: primary school 32.30% (n = 9030), secondary general school 1.93% (n = 539), secondary school 7.73% (n = 2162), secondary academic school 19.50% (n = 5451), comprehensive school 14.36% (n = 4016), special needs school 9.64% (n = 2969), vocational school 9.65% (n = 2699) and other schools 4.89% (n = 1367). Most participants were teachers (94.84%; n = 28,748), 2.00% (n = 605) stated to be a teaching aid, and 3.17% (n = 960) participants were candidates for teaching. Overall, 10.62% (n = 3290) of participants were a member of the school management team, while 89.38% (n = 27,692) stated to be not a member. In case of employment status, 60.28% (n = 18,662) participants indicated to work full-time and 39.72% (n = 12,297) worked part-time.

A total of 27,782 (96.52%) participants switched from face-to-face to ERT between the beginning of the COVID-19 pandemic and the completion of the survey. Overall, 26,667 (88.82%) teachers indicated that they had experienced difficulties in implementing ERT. There were 24,751 (88.16%) teachers who reported that ERT was aggravated by technical difficulties (disconnections, software errors, operational problems) and 22,862 (81.42%) reported an inadequate technical equipment (e.g., nonexistent, or outdated equipment). Technical equipment was most likely missing in students (94.43%), second most likely missing in the school (70.85%), and less likely missing in teachers themselves (31.38%). A total of 17,711 (63.14%) teachers stated to feel overwhelmed with their work-related tasks during ERT.

Overall, teachers partly agreed to have a positive attitude towards ERT with a mean of 3.47 (± .84) on a five-point scale. Teachers stated that they perceived stress from ERT to a great extent with a mean of 5.03 (± .62) on a six-point scale. The attitude towards ERT was negatively correlated with perceived stress from ERT (r = − .15, *p* < .001).

### Group differences by sociodemographic characteristics

Table 1 depicts the mean values and results of the ANOVAs regarding the attitude towards and perceived stress from ERT differentiated by gender, age and number of children living in the household.

**Table 1:** Sociodemographic differences in teachers’ attitude towards and perceived stress from emergency remote teaching (ERT).

	Attitude towards ERT			Perceived stress from ERT		
	Sample, n (%)	Value, mean (SD)	Between-subject factor, F (df), *p*, η^2^	Sample, n (%)	Value, mean (SD)	Between-subject factor, F (df), *p*, η^2^
All	26,117 (100.00)	3.47 (.84)		14,189 (100.00)	5.03 (.62)	
Gender						
(a) Female	20,159 (77.19)	3.46 (.82)	8.54 (2; 239), *p* < .001, < .01^b^	11,422 (80.50)	5.04 (.61)	18.68 (2; 14,186), *p* < .001, < .01^a^
(b) Male	5867 (22.46)	3.51 (.89)		2699 (19.02)	4.96 (.64)	
(c) Diverse	91 (.35)	3.28 (1.08)		68 (.48)	5.06 (.65)	
Age						
(a) 1st quartile	6511 (24.93)	3.63 (.80)	177.28 (3; 14,491), *p* < .001, .02^b^	3377 (23.80)	4.95 (.62)	31.34 (3; 14,185), *p* < .001, < .01^a^
(b) 2nd quartile	6672 (25.55)	3.51 (.83)		3607 (25.42)	5.01 (.60)	
(c) 3rd quartile	6363 (24.36)	3.42 (.86)		3502 (24.68)	5.05 (.62)	
(d) 4th quartile	6571 (25.16)	3.31 (.84)		3703 (26.10)	5.09 (.61)	
Children in household						
(a) 0	13,977 (53.52)	3.45 (.84)	10.74 (3; 24,523), *p* < .001, < .01^a^	7634 (53.80)	5.03 (.62)	1.69 (3; 2801), *p* > .05, .00^b^
(b) 1	4242 (16.24)	3.49 (.83)		2302 (16.22)	5.03 (.62)	
(c) 2	4907 (18.79)	3.53 (.83)		2619 (18.46)	5.00 (.61)	
(d) 3 +	1401 (5.36)	3.48 (.86)		726 (5.12)	5.02 (.59)	

*SD* standard deviation, *p* significance level, *η*^*2*^ eta squared.

^a^ANOVA.

^b^Welch’s T-Test.

#### Attitude towards emergency remote teaching

**Figure 1:**
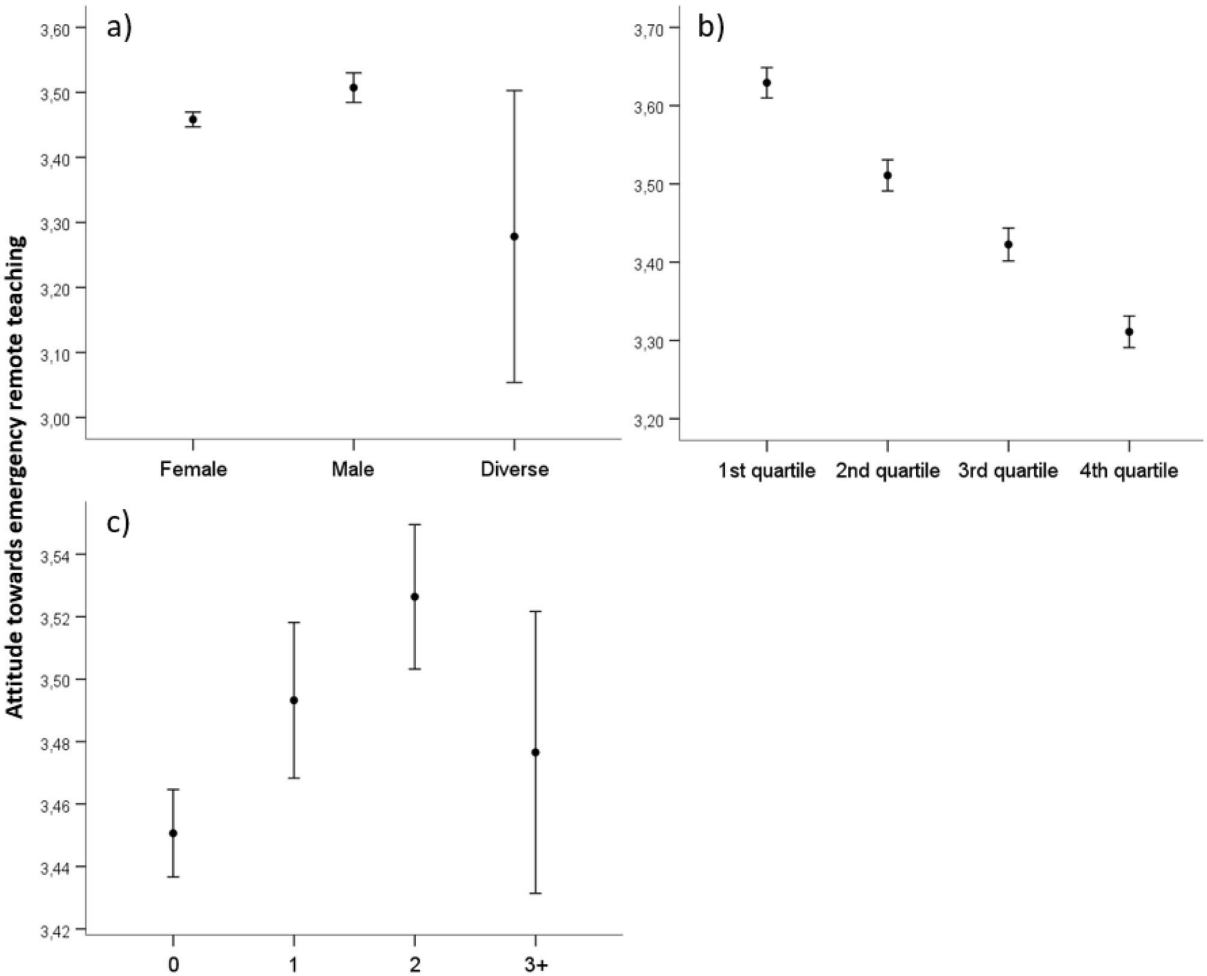
Differences in teachers’ attitude towards emergency remote teaching between (**a**) genders, (**b**) age quartiles, (**c**) number of children living in the household. The first quartile ranges from 18 to 37 years, the second quartile ranges from 38 to 46 years, the third quartile ranges from 47 to 54 years, and the fourth quartile ranges from 55 to 67 years. The circles display the mean and whisker bars display the 95% confidence interval.

Post-hoc analyses revealed significant differences between male and female teachers (*p* ≤ .05), but not between male or female and diverse teachers (*p* > .05). On average, male teachers had a more positive attitude toward ERT than female teachers. There were significant differences between all age quartiles (*p* ≤ .05). The younger the teachers were, the more positive was their attitude towards ERT. Regarding the number of children living in the household, significant differences were revealed between no children, one and two children (*p* ≤ .05). There were no significant differences between one and two children living in the household, and three or more children compared to fewer than three children living in the household (*p* > .05). If there were zero to two children living in the household, the positive attitude of teachers towards ERT increased with the number of children. Figure 1 depicts the differences between gender, age groups, and children living in the household.

#### Perceived stress from emergency remote teaching

**Figure 2:**
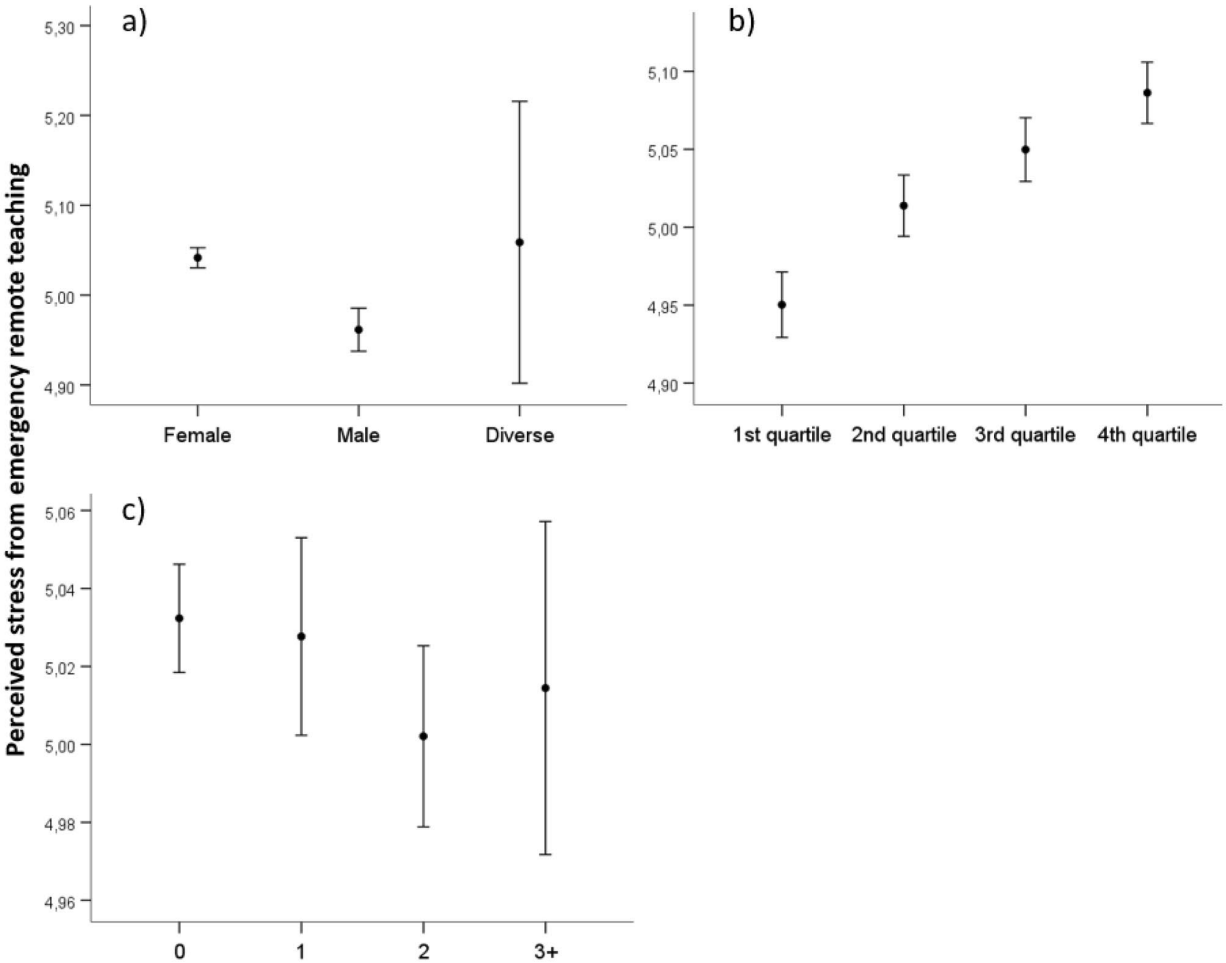
Differences in teachers’ perceived stress from emergency remote teaching between (**a**) genders, (**b**) age quartiles, (**c**) number of children living in the household. The first quartile ranges from 18 to 37 years, the second quartile ranges from 38 to 46 years, the third quartile ranges from 47 to 54 years, and the fourth quartile ranges from 55 to 67 years. The circles display the mean and whisker bars display the 95% confidence interval.

Post-hoc analyses revealed significant differences between male and female teachers (*p* ≤ .05), but not between male or female and diverse teachers (*p* > .05). On average, female teachers felt more stressed from ERT than male teachers. There were significant differences between all age quartiles (*p* ≤ .05), except between the third and the second as well as the third and the fourth quartile (*p* > .05). Overall, the results show that the older the teachers were, the more they felt stressed by ERT. There were no significant differences between teachers with different numbers of children living in their household (*p* > .05). Figure 2 depicts the differences between gender, age groups, and children living in the household.

### Group differences by work-related characteristics

Table 2 shows the mean values and results of the ANOVAs regarding the attitude towards and perceived stress from ERT differentiated by work-related characteristics.

**Table 2:** Work-related differences in teachers’ attitude towards and perceived stress from emergency remote teaching (ERT).

	Attitude towards ERT			Perceived stress from ERT		
	Sample, n (%)	Value, mean (SD)	Between-subject factor, F (df), *p*, η^2^	Sample, n (%)	Value, mean (SD)	Between-subject factor, F (df), *p*, η^2^
All	26,117 (100.00)	3.47 (.84)		14,189 (100.00)	5.03 (.62)	
School type						
(a) Primary school	7490 (31.87)	3.47 (.83)	.93 (7; 23,494), *p* > .05, .00^a^	4091 (31.94)	5.01 (.62)	.94 (7; 12,802), *p* > .05, .00^a^
(b) Secondary general school	452 (1.92)	3.47 (.85)		243 (1.90)	5.06 (.62)	
(c) Secondary school	1802 (7.67)	3.50 (.84)		1020 (7.96)	5.03 (.63)	
(d) Secondary academic school	4647 (19.77)	3.47 (.84)		2503 (19.54)	5.02 (.60)	
(e) Comprehensive school	3361 (14.30)	3.47 (.83)		1847 (14.42)	5.04 (.61)	
(f) Special needs school	2279 (9.70)	3.49 (.85)		1247 (9.73)	5.01 (.60)	
(g) Vocational school	2314 (9.85)	3.48 (.85)		1218 (9.51)	5.05 (.61)	
(h) Others	1157 (4.92)	3.42 (.84)		641 (5.00)	5.03 (.64)	
Occupational group						
(a) Teacher	24,452 (93.62)	3.47 (.84)	23.58 (2; 705), *p* < .001, < .01^b^	13,349 (94.08)	5.03 (.62)	2.18 (2; 13,916), *p* > .05, .00^a^
(b) Teaching aid	387 (1.48)	3.28 (.83)		156 (1.10)	5.03 (.56)	
(c) Candidate	737 (2.82)	3.62 (.80)		414 (2.92)	4.96 (.61)	
School management						
(a) Yes	2820 (10.80)	3.63 (.81)	122.04 (1; 3598), *p* < .001, < .01^b^	1334 (9.40)	5.01 (.63)	1.55 (1; 14,142), *p* > .05, .00^a^
(b) No	23,221 (88.91)	3.45 (.84)		12,810 (90.28)	5.03 (.61)	
Employment						
(a) Full-time	15,828 (60.60)	3.51 (.85)	82.97 (1; 22,293), *p* < .001, < .01^b^	8481 (59.77)	5.04 (.62)	15,78 (1; 12,392), *p* < .001, < .01^b^
(b) Part-time	10,194 (39.03)	3.41 (.82)		5666 (39.93)	5.00 (.60)	

*SD* standard deviation, *p* significance level, *η*^*2*^ eta squared.

^a^ANOVA.

^b^Welch’s T-Test.

#### Attitude towards emergency remote teaching

**Figure 3:**
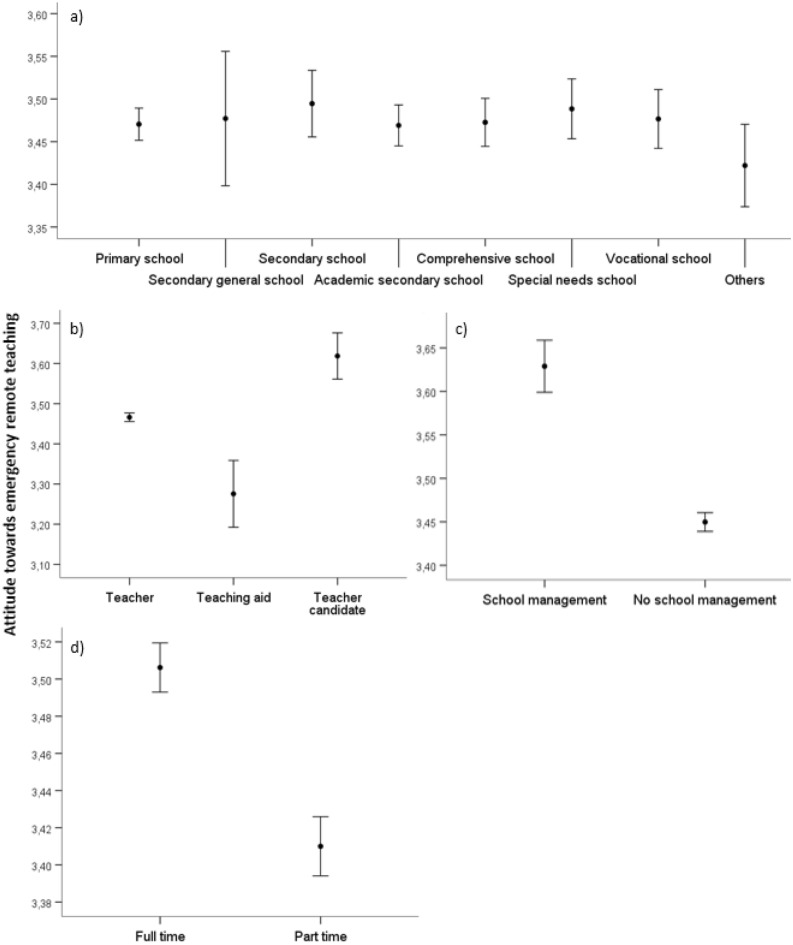
Differences in teachers’ attitude towards emergency remote teaching between (**a**) German school types, (**b**) occupational status, (**c**) members and non-members of school management, (**d**) employment status. The circles display the mean and whisker bars display the 95% confidence interval.

There were no differences between the different school types (*p* > .05) but between all occupational groups: Teaching candidates had the most positive attitude towards ERT compared to teachers and teaching aids, who had the least positive attitude. Members of the school management showed a significantly more positive attitude towards ERT than non-members (*p* ≤ .05). Full-time teachers revealed a significantly more positive attitude towards ERT than part-time teachers (*p* ≤ .05). Figure 3 depicts the differences between school types, occupational status, members and non-members of school management, and employment status.

#### Perceived stress from emergency remote teaching

**Figure 4:**
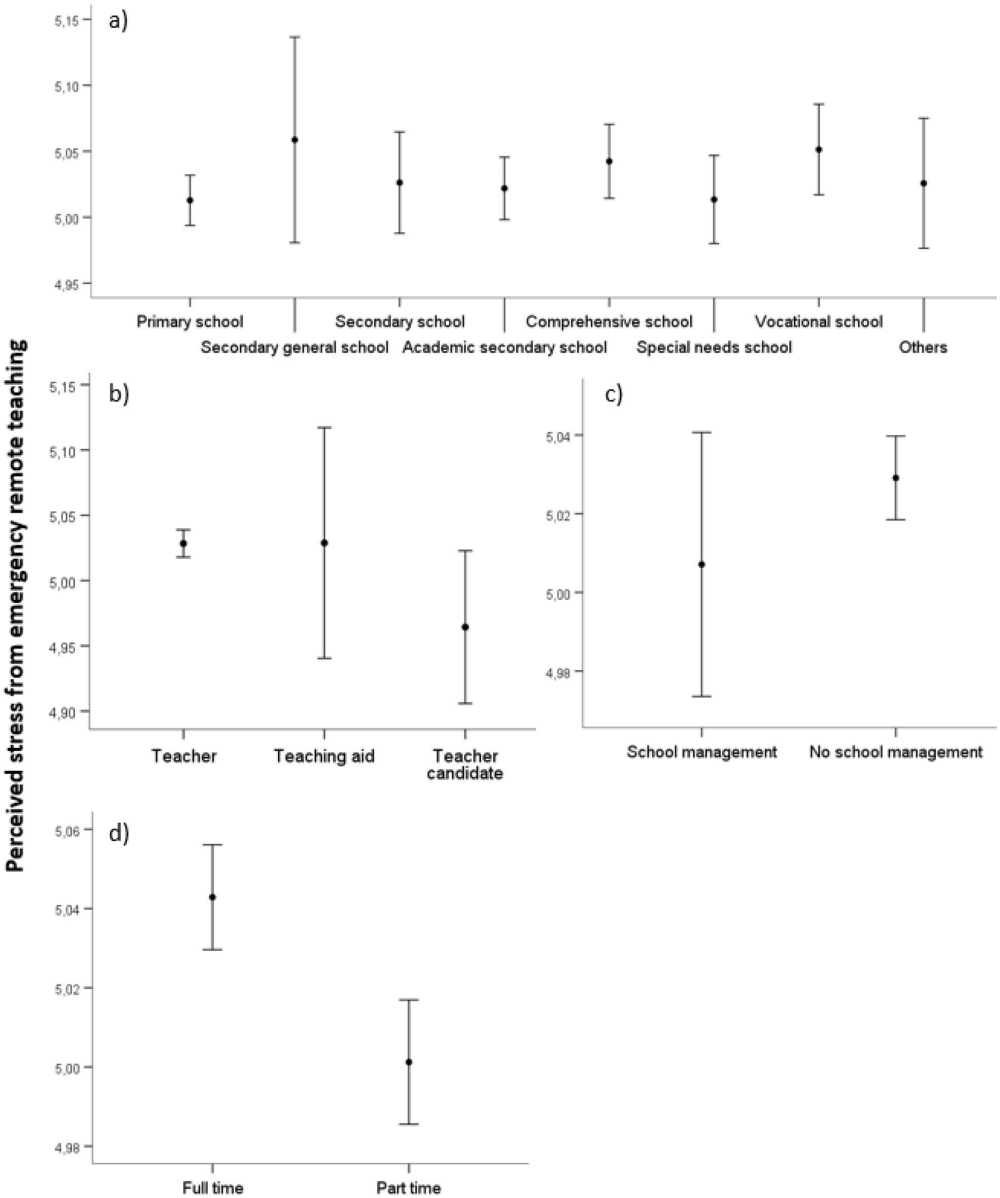
Differences in teachers’ perceived stress from emergency remote teaching between (**a**) German school types, (**b**) occupational status, (**c**) members and non-members of school management, (**d**) employment status. The circles display the mean and whisker bars display the 95% confidence interval.

There were no significant differences between school types (*p* > .05). There were no significant differences between the occupational groups or school management membership compared to no membership (*p* > .05). Significant differences were found between part-time and full-time teachers (*p* ≤ .05). Full-time teachers perceived, on average, more stress from ERT compared to part-time teachers. Figure 4 depicts the differences between school types, occupational status, members and non-members of school management, and employment status.

## Discussion

Overall, teachers’ attitude towards ERT tended to be positive, although perceived stress was high. Other research from Germany showed comparable results^3,10,12^. The present study demonstrates that both, the attitude towards and the perceived stress from ERT among German school teachers, differed regarding sociodemographic and work-related variables. The results are mostly in line with previous results from Drossel et al.^11^ investigating important predictors for the successful implementation of digital media in German schools before the COVID-19 pandemic. In our study, however, there may have been a ceiling effect on teachers’ experience of stress: Because the overall stress perceived by teachers was already relatively high^3^, differences between subgroups might remain small. In other words, when the stress levels of the participants are all clustered near the highest possible score (the “ceiling”), the analysis could lose power.

It can be summarized, that teachers who had a more positive attitude towards ERT felt less stressed from it, which may contribute to a more effective implementation of remote teaching in schools. This is consistent with the findings of Košir et al.^13^, who showed that the attitude towards ERT is a significant predictor of teachers’ experienced stress levels.

### Sociodemographic differences

The proportion of women in our study population is relatively high (77.0%) but representative of the gender distribution among teachers in Germany, where women accounted for 73.4% of all teachers^33^. As hypothesized, male teachers showed a more positive attitude towards and less perceived stress from ERT than female teachers (see “Introduction” Section, para. 7). Studies on a gender dependent frequency of digital media use are inconsistent and are described as country-dependent^34^. In Germany and Greece, male teachers are more likely to use digital media in the classroom than female teachers^27,35^. The reason for a more frequent use and more positive attitude towards the implementation of digital media in school in male teachers is often explained by men’s greater involvement in technology in general^16,27,35,36^. Moreover, previous studies reported a higher perceived self-efficacy as well as digital skill level in men^16,25^. The stereotype that information and communication technology is defined as more masculine and related to mathematical, technical, and logical skills might compound this gender difference^16^. Additionally, studies that were performed during the pandemic indicate higher levels of COVID-19-generated stress and mental burdens in women compared to men^35–37^. High stress levels might hinder female teachers’ willingness to implement remote teaching even more.

As expected, the positivity of attitude towards ERT was lower in older age groups, while the perceived stress from ERT was higher (see “Introduction” Section, para. 7). The influence of age on the attitude of teachers using digital media for teaching is controversially discussed in the current literature. Some studies revealed no or just a little age effect^34,38,39^, whereas others described age as a significant mediator in affecting participants’ attitude towards remote teaching, even before the pandemic^40^. In accordance with our results, in Germany, younger teachers seem to have a more enthusiastic attitude towards the use of information and communication technologies for teaching compared to older teachers^9^. This could be due to a different socialization of younger generations with digital media.

Many teachers not only had to deal with ERT of their students, but also of their own children. Therefore, teachers taking care of their own children were identified as being more vulnerable to stress^41^ and perceived higher stress levels by working from home^13^. Therefore, we expected teachers with more children living in the own household to experience a more negative attitude towards and more stress from ERT (see “Introduction” Section, para. 7). We obtained no differences regarding perceived stress between teachers with a different number of own children. However, the more children living in the household (from zero up to two children) the more positive was the teachers’ attitude towards ERT. One possible explanation could be that children provide teacher parents with easier access to and assistance with remote teaching, since children nowadays often learn to use digital media at an early age^42^. Since taking care of own children can cause additional stress, better options for day care of teachers’ children should be considered (in the case of working from home again)^43^.

### Work-related differences

The evidence regarding work-related differences in attitude towards and perceived stress from ERT seems inconsistent. This is the first study taking all school types into consideration. In contrast to our expectations, we revealed no differences between school types (see “Introduction” Section, para. 7). Another study from Germany performed right before the pandemic showed that teachers who worked in higher tracks of education showed a more positive attitude towards remote teaching and perceived less stress from it compared to teachers from lower tracks of education^27^. Moreover, Dincher and Wagner^44^ reported that teachers in elementary schools in Germany used mainly established technologies (paper-based assignments, phone calls, and emails) while secondary teachers predominantly used learning platforms and e-mails for ERT. Jelińska and Paradowski^36^ reported that teachers in higher educational tracks or with private tuition/self-employees/freelancers showed higher engagement in online teaching and better coping strategies with ERT during COVID-19.

In our study, the full-time teachers stated a more positive attitude towards ERT but felt also more stressed from it. There were no differences between occupational groups regarding perceived stress, but teaching candidates had a more positive attitude towards ERT compared to teachers and teaching aids, who had the least positive attitude. Papazis et al.^35^ did not detect a significant difference between teachers holding a permanent post and their temporary colleagues regarding levels of stress, although the resilience level was higher in permanent teachers. A possible explanation might be that while teachers see an opportunity in remote teaching in general, the spontaneity of the transition had led to an increased workload due to a lack of technology and skills as well as a permanent reachability to students and parents. This may be more difficult for full-time teachers to compensate for and could in turn lead to a poor work-life balance. Members of the school management showed a more positive attitude towards ERT than non-members, but the stress level did not differ between groups. Further studies are needed, which examine in more detail the difference in the attitude towards ERT between school management and teachers, particularly as many teachers take on school management tasks.

### Practical implications

In consideration of the state of research on the digitalization of the German school system, it seems appropriate to further promote, raise awareness, and support it in general. In this context, it can help to focus on particular groups when implementing (emergency) remote teaching. Female and older teachers seem to have a more negative attitude towards and perceive a little more stress from ERT. Studies suggested that this may be due to lower self-efficacy and digital skills in women compared to men or in terms of age, through less experience in using digital media in everyday life. Thus, special attention should be paid to the training of digital competencies among female and older teachers. This could also reduce the higher stress levels associated with ERT. In this context, the work-life balance of teachers should receive special attention, for example by providing day-care for teachers’ own children, fixed working hours and/or education on resilience and coping strategies. Additionally, further investigation of the school types influencing teachers’ attitude towards and perceived stress from (emergency) remote teaching seems worthwhile. Focusing on students with learning difficulties or less independence in learning (e.g., special school, general school) as well as the size of classes could be important.

The adoption and implementation of (emergency) remote teaching and using digital media in school depends on teachers’ beliefs in effective learning through digital media and their attitude towards as well as their (technical) skills and their self-efficacy in remote teaching. Accordingly, policy-makers and schools should not limit their efforts on the provision of the necessary technologies, but should invest in target group specific training and information on how to use these technologies to ensure that teachers are less stressed by this new norm of teaching^45^.

### Limitations

Our study was carried out in Germany, therefore generalization of the results to other countries and their educational contexts is, especially regarding the different levels of digitalization in schools between countries, hardly possible.

At the time of the survey (March 2021), Germany was at the beginning of the “third corona wave” with a sharp rise in the number of COVID-19 infections. This suggests that the participants were confronted with increased uncertainties at the time of the survey, as it was not possible to predict how long remote teaching or restrictions in the school environment would last. In addition, it is unclear what biases may have operated in the course of recruiting participants. For example, whether it was primarily those teachers who were particularly burdened who participated in the survey or, conversely, those who were not too burdened and, thus, still had time and energy to answer all questions. In addition, the level of education of the teachers might have had an impact on the attitude towards remote teaching as well as the perceived stress from ERT. The level of education should be taken into account in follow-up studies.

The questionnaire was very long (with 353 items) and took an average time of 35 min to complete, which could have caused a risk of participant fatigue and might have resulted in fewer or inaccurate responses. About 16.00% of the included participants answered the questions on attitude towards ERT and 54.36% the questions on perceived stress from ERT incompletely or incorrectly. Follow-up studies should consider this risk. Furthermore, the survey instruments regarding the attitude towards and the perceived stress from ERT had to be developed completely new, which may have limited their validity. Moreover, the low level of the Cronbach’s α for the perceived stress item (α = .66) might indicate that emergency remote teaching was not fundamentally perceived as stressful, but that certain aspects of it (e.g., lack of equipment or technical problems) caused more or less stress for the teachers.

## Conclusion

Overall, teachers indicated a rather positive attitude towards ERT, although perceived stress was high. A more positive attitude towards ERT seems to be associated with lower stress levels. Sociodemographic characteristics like being female, higher age, and higher number of children living in the own household as well as a work-related characteristic like full-time employment might hinder an effective implementation of (emergency) remote teaching in school settings in Germany. Policy-makers and schools should think of strategies to improve the attitude towards and decrease perceived stress from (emergency) remote teaching. One possibility could be subgroup-specific training on the use of digital media, adapted to the work environment. Future studies should validate our findings during the course of the pandemic and afterwards, especially regarding work-related factors. Additional research could examine the feasibility and success rate of used strategies with regard to the attitude towards and higher stress levels caused by ERT.

### Supplementary Information

Supplementary Information.

## Data Availability

The datasets used and/or analyzed during the current study are available from the corresponding author on reasonable request. The data are not publicly available due to privacy restrictions.
