# Supplementary material for: Sociodemographic and work-related differences in teachers’ attitude towards and perceived stress from emergency remote teaching during the COVID-19 pandemic
Source: Sci Rep. 2023 Aug 10;13:12999. doi: 10.1038/s41598-023-39824-w (PMC10415343; doi:10.1038/s41598-023-39824-w)
Supplement: Supplementary file 1 — Supplementary Information. [file 41598_2023_39824_MOESM1_ESM.pdf]

Fragebogen

SARS-CoV-2 Arbeits- und Infektionsschutzmaßnahmen an Schulen

Sehr geehrte Damen und Herren,

wir bedanken uns für Ihre Bereitschaft, an der vorliegenden bundesweiten Studie **SARS-CoV-2 Arbeits- und Infektionsschutzmaßnahmen an Schulen** teilzunehmen. Es handelt sich hierbei um ein Forschungsprojekt, welches von der Universitätsmedizin Mainz im Auftrag der Bundesanstalt für Arbeitsschutz und Arbeitsmedizin (BAuA) durchgeführt wird.

Für uns alle stellt die COVID-19-Pandemie eine immense Herausforderung dar. Ziel ist es daher, herauszufinden, wie sich die Ausbreitung der Pandemie und die zur Eindämmung getroffenen Maßnahmen speziell auf die **Lebenswelt Schule** auswirken. Mit Ihrer Teilnahme an dieser Umfrage leisten Sie einen wichtigen Beitrag zur Erforschung von Belastungen und Beanspruchungen von Lehrkräften an Schulen während der COVID-19-Pandemie. Beispielsweise sollen der Umgang mit Hygieneplänen und organisatorischen / pädagogischen Herausforderungen im Schulbetrieb erfasst werden. Sie tragen dazu bei, anhand der erhobenen Daten Empfehlungen für Schulen und Lehrkräfte ableiten zu können. Dafür danken wir Ihnen!

Im Rahmen unserer Studie spenden wir 2.000,00 € an das Projekt „Corona-Nothilfe-Pakete“ des Deutschen Kinderhilfswerk e.V.

Informationen zum Verwendungszweck der Spende finden Sie unter: https://www.dkhw.de/foerderung/corona-nothilfe-pakete/

Die Teilnahme an der Umfrage sowie die Beantwortung der einzelnen Fragen sind **freiwillig**, durch die Nicht-Bearbeitung entstehen Ihnen keinerlei Nachteile. Ihre Daten werden **vollständig anonym** erfasst und verschlüsselt übertragen. Eine Zuordnung der Daten zu einer Person ist somit nicht möglich. Die Umfrage beinhaltet Fragen zu Ihrem schulischen und privaten Umfeld. Die Bearbeitung wird insgesamt etwa 30 Minuten dauern. Bestätigen Sie bitte durch das Setzen eines Hakens neben dem Hinweis zur Datenschutzerklärung, dass Sie mindestens 18 Jahre alt sind, diese Erklärung gelesen haben, der Verarbeitung besonderer Kategorien personenbezogener Daten (nach Art. 9 DSGVO) zustimmen und an der Umfrage teilnehmen möchten.

☐ Ich habe die Hinweise zum Datenschutz gelesen und stimme der Verarbeitung meiner Daten zu

0. Pseudonymisierungscode

PC1	Wie lautet der erste Buchstabe des Vornamens Ihrer Mutter?			
PC2	Wie lautet der erste Buchstabe des Vornamens Ihres Vaters?			
PC3	Wie lautet der letzte Buchstabe des Geburtsmonats Ihrer Mutter?			
PC4	Wie lautet der letzte Buchstabe des Geburtsmonats Ihres Vaters?			
PC5	Wie lautet die letzte Ziffer des Geburtsjahres Ihrer Mutter?			
PC6	Wie lautet die letzte Ziffer des Geburtsjahres Ihres Vaters?			

Hilfstext

Beispiele:

Erster Buchstabe des Vornames der Mutter: Uta = **U**

Letzter Buchstabe des Geburtsmonats der Mutter: Februar= **r**

Letzte Ziffer des Geburtsjahres Ihrer Mutter: 1958 = **8**

1. Soziodemografische Variablen & Angaben zum Arbeitsplatz

Soziodemografie

A1	Geschlecht	☐ weiblich	☐ männlich	☐ divers
A2	Alter (in Jahren)	z. B. 35		
A3	Anzahl der Personen in Ihrem Haushalt?<br><br>(Hinweistext: Bitte zählen Sie sich hierbei mit.)	z. B. 3		
A4	Davon minderjährige Kinder im eigenen Haushalt lebend	z. B. 3		
SW1	(Filterfrage, wenn A1 = weiblich ODER divers)<br><br>Sind sie aktuelle schwanger?	☐ ja	☐ nein	☐ keine Antwort

Angaben zum Arbeitsplatz

Im folgenden Abschnitt geht es um schulbezogene Angaben.

A5	An welcher Schulart sind Sie tätig?<br>(Mehrfachnennung möglich)	☐ Grundschule	☐ Hauptschule	☐ Realschule	☐ Realschule plus<br>(Kombination aus Haupt- und Realschule)	☐ Integrierte Gesamtschule<br>(Kombination aus Haupt- und Realschule sowie Gymnasium)
		☐ Gymnasium	☐ Förderschule	☐ Berufsschule	☐ Andere Schulart [Freitext]	
A6	Welcher Berufsgruppe gehören Sie an?	☐ Lehrkraft	☐ Pädagogische Fachkraft / Unterrichtshilfe / Assistent*in o.ä.	☐ Anwärter*in	☐ Sonstige<br>[Freitext]	
A7	Sind Sie Teil des Schulleitungsteams?	☐ ja	☐ nein			
A8	Beschäftigungsverhältnis	☐ verbeamtet	☐ beschäftigt, unbefristeter Vertrag	☐ beschäftig, befristeter Vertrag	☐ Sonstiges	
A9	Arbeitszeitmodell	☐ Vollzeit	☐ Teilzeit			
A10	Welche Fächer unterrichten Sie?<br>(Hinweistext: Bitte geben Sie jeweils ein Fach je Freitextfeld ein)	z. B. Mathematik	z. B. Deutsch			
		z. B. Sport	z. B. Englisch			
		z. B. Sport	z. B. Musik			
A11	Wie viele Klassen unterrichten Sie?	z. B. 4				
A12	Welche Klassenstufen unterrichten Sie aktuell?	Von	z. B. 1	bis	z. B. 13	

A13	In welchem Bundesland befindet sich Ihre Dienststelle?	Dropdown 16 Bundesländer	
A14	Arbeiten Sie an mehr als einer Dienststelle (z. B. zwei Schulen oder Schule und Studienseminar)?	☐ ja	☐ nein
A15	Gibt es Betreuungssituationen, die engen Schülerkontakt mit sich bringen (z. B. Ganztagsschule, AGs, Vertrauenslehrer*in)?	☐ ja	☐ nein
A16	Sind Sie in die Pflege von Schüler*innen involviert (z.B. an einer Förderschule)?	☐ ja	☐ nein

2. Identifizierung von SARS-CoV-2-spezifischen Belastungen und Herausforderungen

Die nachfolgenden Aussagen und Fragen beziehen sich auf die Zeit seit dem Ausbruch der COVID-19-Pandemie sowie die damit verbundenen Maßnahmen zum Infektionsschutz in Deutschland, also den Zeitraum seit etwa März 2020.

2a) Organisatorisch

Im folgenden Abschnitt geht es um die besonderen organisatorischen Belastungen und Herausforderungen, die sich für Sie im Schulbetrieb während der COVID-19-Pandemie ergeben haben können (denken Sie dabei bitte sowohl an Phasen des Distanzunterrichts als auch an Phasen des Präsenzunterrichts).

Bitte beantworten Sie die Fragen spontan. Es gibt keine „falschen Antworten“, was immer Ihnen zutreffend erscheint, ist richtig.

2a)							
**Erleben Sie aufgrund der COVID-19-Pandemie Veränderungen in nachfolgend genannten Bereichen im Vergleich zur Zeit vor der COVID-19-Pandemie?**							
CAO1	Schulorganisatorische Prozesse im Allgemeinen	Ja ☐			Nein ☐		
CAO1a	(Filterfrage, falls „Ja“)<br>Empfinden Sie diese Veränderung als belastend?	In sehr hohem Maße	in hohem Maße	zum Teil	in geringem Maße	in sehr geringem Maße	gar nicht
		☐	☐	☐	☐	☐	☐

2a)							
CAO2	Stundenplangestaltung (z. B. Vorlaufzeit, Vollständigkeit oder Klarheit)	Ja ☐			Nein ☐		
CAO2a	(Filterfrage, falls „Ja“)<br>Empfinden Sie diese Veränderung als belastend?	In sehr hohem Maße	in hohem Maße	zum Teil	in geringem Maße	in sehr geringem Maße	gar nicht
		☐	☐	☐	☐	☐	☐
CAO3	Einschränkungen bei der Raumnutzung (z. B. Platzmangel)	Ja ☐			Nein ☐		
CAO3a	(Filterfrage, falls „Ja“)<br>Empfinden Sie diese Veränderung als belastend?	In sehr hohem Maße	in hohem Maße	zum Teil	in geringem Maße	in sehr geringem Maße	gar nicht
		☐	☐	☐	☐	☐	☐
CAO4	Pausengestaltung (z. B. getrennte Kleingruppen, Veränderung der Anzahl an Pausenaufsichtspersonen)	Ja ☐			Nein ☐		
CAO4a	(Filterfrage, falls „Ja“)<br>Empfinden Sie diese Veränderung als belastend?	In sehr hohem Maße	in hohem Maße	zum Teil	in geringem Maße	in sehr geringem Maße	gar nicht
		☐	☐	☐	☐	☐	☐
CAO5	Möglichkeit zum Austausch mit Kolleg*innen	Ja ☐			Nein ☐		
CAO5a	(Filterfrage, falls „Ja“)<br>Empfinden Sie diese Veränderung als belastend?	In sehr hohem Maße	in hohem Maße	zum Teil	in geringem Maße	in sehr geringem Maße	gar nicht
		☐	☐	☐	☐	☐	☐

2a)							
CAO6	Zusammenarbeit mit Kolleg*innen (z. B. Absprachen oder zur Unterrichtsvorbereitung)	Ja ☐			Nein ☐		
CAO6a	(Filterfrage, falls „Ja“)<br>Empfinden Sie diese Veränderung als belastend?	In sehr hohem Maße	in hohem Maße	zum Teil	in geringem Maße	in sehr geringem Maße	gar nicht
		☐	☐	☐	☐	☐	☐
CAO7	Gegenseitiges Bereitstellen von Arbeits- und Unterrichtsmaterialien im Kollegium	Ja ☐			Nein ☐		
CAO7a	(Filterfrage, falls „Ja“)<br>Empfinden Sie diese Veränderung als belastend?	In sehr hohem Maße	in hohem Maße	zum Teil	in geringem Maße	in sehr geringem Maße	gar nicht
		☐	☐	☐	☐	☐	☐
CAO8	Erhalt aller Informationen, die Sie für die Ausübung Ihrer Tätigkeit benötigen	Ja ☐			Nein ☐		
CAO8a	(Filterfrage, falls „Ja“)<br>Empfinden Sie diese Veränderung als belastend?	In sehr hohem Maße	in hohem Maße	zum Teil	in geringem Maße	in sehr geringem Maße	gar nicht
		☐	☐	☐	☐	☐	☐
CAO9	Zunahme der Informationsmenge für schulische Belange (z. B. E-Mails, Messenger, Aushänge, Mündliches)	Ja ☐			Nein ☐		

2a)							
CAO9a	(Filterfrage, falls „Ja“)<br>Empfinden Sie diese Veränderung als belastend?	In sehr hohem Maße	in hohem Maße	zum Teil	in geringem Maße	in sehr geringem Maße	gar nicht
		☐	☐	☐	☐	☐	☐
CAO10	Konferenzen und Dienstbesprechungen	Ja ☐			Nein ☐		
CAO10a	(Filterfrage, falls „Ja“)<br>Empfinden Sie diese Veränderung als belastend?	In sehr hohem Maße	in hohem Maße	zum Teil	in geringem Maße	in sehr geringem Maße	gar nicht
		☐	☐	☐	☐	☐	☐
CAO11	Klarheit bezüglich Ihres Verantwortungsbereichs	Ja ☐			Nein ☐		
CAO11a	(Filterfrage, falls „Ja“)<br>Empfinden Sie diese Veränderung als belastend?	In sehr hohem Maße	in hohem Maße	zum Teil	in geringem Maße	in sehr geringem Maße	gar nicht
		☐	☐	☐	☐	☐	☐
CAO12	Mehr emotionale Anforderungen der Arbeit<br>(z B. Verbergen von Gefühlen)	Ja ☐			Nein ☐		
CAO12a	(Filterfrage, falls „Ja“)<br>Empfinden Sie diese Veränderung als belastend?	In sehr hohem Maße	in hohem Maße	zum Teil	in geringem Maße	in sehr geringem Maße	gar nicht
		☐	☐	☐	☐	☐	☐
CAO13	Erhöhung der Arbeitsmenge	Ja ☐			Nein ☐		

2a)								
CAO13a	(Filterfrage, falls „Ja“)<br>Empfinden Sie diese Veränderung als belastend?	In sehr hohem Maße<br>☐	in hohem Maße<br>☐	zum Teil<br>☐	in geringem Maße<br>☐	in sehr geringem Maße<br>☐	gar nicht<br>☐
CAO14	Gerechte Konfliktlösung durch die Schulleitung	Ja ☐			Nein ☐		
CAO14a	(Filterfrage, falls „Ja“)<br>Empfinden Sie diese Veränderung als belastend?	In sehr hohem Maße<br>☐	in hohem Maße<br>☐	zum Teil<br>☐	in geringem Maße<br>☐	in sehr geringem Maße<br>☐	gar nicht<br>☐
CAO15	Gerechte Verteilung der Arbeit	Ja ☐			Nein ☐		
CAO15a	(Filterfrage, falls „Ja“)<br>Empfinden Sie diese Veränderung als belastend?	In sehr hohem Maße<br>☐	in hohem Maße<br>☐	zum Teil<br>☐	in geringem Maße<br>☐	in sehr geringem Maße<br>☐	gar nicht<br>☐
CAO16	Anerkennung und Wertschätzung Ihrer Arbeit durch die Schulleitung	Ja ☐			Nein ☐		
CAO16a	(Filterfrage, falls „Ja“)<br>Empfinden Sie diese Veränderung als belastend?	In sehr hohem Maße<br>☐	in hohem Maße<br>☐	zum Teil<br>☐	in geringem Maße<br>☐	in sehr geringem Maße<br>☐	gar nicht<br>☐

2b) Pädagogisch

Im folgenden Abschnitt geht es um die besonderen pädagogischen Belastungen und Herausforderungen, die sich für Sie im Schulbetrieb während der COVID-19-Pandemie ergeben haben können (denken Sie dabei bitte sowohl Phasen des Distanzunterrichts als auch Phasen des Präsenzunterrichts).

Bitte beantworten Sie die Fragen spontan. Es gibt keine „falschen Antworten", was immer Ihnen zutreffend erscheint, ist richtig.

Pädagogische Belastungen und Herausforderungen für Lehrkräfte

2b)							
**Erleben Sie aufgrund der COVID-19-Pandemie:**							
CAP1	Probleme bei der Umsetzung des Bildungsauftrags	Ja ☐			Nein ☐		
CAP1a	(Filterfrage, falls „Ja")<br>Empfinden Sie diese Veränderung als belastend?	In sehr hohem Maße	in hohem Maße	zum Teil	in geringem Maße	in sehr geringem Maße	gar nicht
		☐	☐	☐	☐	☐	☐
CAP2	Notwendigkeit zur Änderung der Didaktik des Präsenzunterrichts	Ja ☐			Nein ☐		
CAP2a	(Filterfrage, falls „Ja")<br>Empfinden Sie diese Veränderung als belastend?	In sehr hohem Maße	in hohem Maße	zum Teil	in geringem Maße	in sehr geringem Maße	gar nicht
		☐	☐	☐	☐	☐	☐

2b)							
CAP3	Schwierigkeiten bei der Umsetzung von Distanzunterricht	Ja ☐			Nein ☐		
CAP3a	(Filterfrage, falls „Ja“)<br>Empfinden Sie diese Veränderung als belastend?	In sehr hohem Maße	in hohem Maße	zum Teil	in geringem Maße	in sehr geringem Maße	gar nicht
		☐	☐	☐	☐	☐	☐

Belastungen und Herausforderungen bei der Umsetzung des Bildungsauftrages

2c)							
**Erleben Sie aufgrund der COVID-19-Pandemie:**							
CAP4	Eine Verschlechterung der <u>schulischen</u> Entwicklung Ihrer Schüler*innen	Ja ☐			Nein ☐		
CAP4a	(Filterfrage, falls „Ja“)<br>Empfinden Sie diese Veränderung als belastend?	In sehr hohem Maße	in hohem Maße	zum Teil	in geringem Maße	in sehr geringem Maße	gar nicht
		☐	☐	☐	☐	☐	☐
CAP5	Eine Verschlechterung der <u>persönlichen</u> Entwicklung Ihrer Schüler*innen	Ja ☐			Nein ☐		
CAP5a	(Filterfrage, falls „Ja“)<br>Empfinden Sie diese Veränderung als belastend?	In sehr hohem Maße	in hohem Maße	zum Teil	in geringem Maße	in sehr geringem Maße	gar nicht
		☐	☐	☐	☐	☐	☐

2c)							
CAP6	Einen schlechteren Kontakt zu Ihren Schüler*innen	Ja ☐			Nein ☐		
CAP6a	(Filterfrage, falls „Ja“)<br>Empfinden Sie diese Veränderung als belastend?	In sehr hohem Maße	in hohem Maße	zum Teil	in geringem Maße	in sehr geringem Maße	gar nicht
		☐	☐	☐	☐	☐	☐
CAP7	Einen schlechteren Kontakt zu den Erziehungsberechtigten Ihrer Schüler*innen	Ja ☐			Nein ☐		
CAP7a	(Filterfrage, falls „Ja“)<br>Empfinden Sie diese Veränderung als belastend?	In sehr hohem Maße	in hohem Maße	zum Teil	in geringem Maße	in sehr geringem Maße	gar nicht
		☐	☐	☐	☐	☐	☐
CAP8	Höhere Erwartungen der Erziehungsberechtigten Ihrer Schüler*innen an Ihre Arbeit	Ja ☐			Nein ☐		
CAP8a	(Filterfrage, falls „Ja“)<br>Empfinden Sie diese Veränderung als belastend?	In sehr hohem Maße	in hohem Maße	zum Teil	in geringem Maße	in sehr geringem Maße	gar nicht
		☐	☐	☐	☐	☐	☐
CAP9	Schwierigkeiten beim Erreichen der angestrebten Lernziele mit Ihren Schüler*innen	Ja ☐			Nein ☐		
	(Filterfrage, falls „Ja“)	In sehr hohem Maße	in hohem Maße	zum Teil	in geringem Maße	in sehr geringem Maße	gar nicht

2c)							
CAP9a	Empfinden Sie diese Veränderung als belastend?	☐	☐	☐	☐	☐	☐

2d) Umstellung auf digitale Unterrichtsformate / Homeschooling

An vielen Schulen in Deutschland gab es während der COVID19-Pandemie eine stärkere Nutzung von digitalen Unterrichtsformaten. Im folgenden Abschnitt geht es um die besonderen Belastungen und Herausforderungen, die sich für Sie im Schulbetrieb während der COVID-19-Pandemie ergeben haben können.

Bitte beantworten Sie die Fragen spontan. Es gibt keine „falschen Antworten“, was immer Ihnen zutreffend erscheint, ist richtig.

Distanzunterricht

2d)							
CAD1	Haben Sie Ihren Unterricht während der COVID-19-Pandemie (phasenweise) von Präsenzunterricht auf Distanzunterricht umgestellt?	☐ Ja			☐ Nein		
CAD1a	(Filterfrage, falls „Ja“)<br>Empfinden Sie diese Veränderung als belastend?	In sehr hohem Maße<br>☐	in hohem Maße<br>☐	zum Teil<br>☐	in geringem Maße<br>☐	in sehr geringem Maße<br>☐	gar nicht<br>☐
CAD2	In welchem Modus unterrichten Sie aktuell?	☐ Präsenzunterricht		☐ Distanzunterricht		☐ Beide Formen	
CAD3	*(Filterfragen folgend, falls CAD1 = Ja ODER CAD2= Distanzunterricht ODER CAD2=Beide Formen, dann CAD3-CAD8 anzeigen)*<br>Welche Formen der Unterstützung haben Sie Ihren Schüler*innen während des Distanzunterrichts angeboten?	☐ Online-Lernplattformen (z.B. Moodle)	☐ Videos aus dem Internet	☐ selbst erstellte Videos	☐ Papierausdruck (Download)	☐ Papierausdruck (per Post oder zur Abholung)	
		☐ Internetseiten	☐ E-Mail-Kontakte	☐ Messenger-Kontakte	☐ Telefonate	☐ gemeinsamer webbasierter Unterricht (z. B. per Videoanruf)	

	(Mehrfachnennungen möglich)	☐ persönliche Gespräche	☐ Präsentationen	☐ Apps		
CAD4	Wie häufig tauschten Sie sich durchschnittlich mit Ihren Schüler*innen während des Distanzunterrichts aus?	☐ mehrmals täglich	☐ einmal täglich	☐ mehrmals pro Woche	☐ einmal pro Woche	☐ weniger als einmal pro Woche	☐ fast nie / nie
CAD5	Der Distanzunterricht wurde von technischen Problemen erschwert (z. B. Verbindungsabbrüche, Softwarefehler, Probleme bei der Bedienung).	Ja ☐			Nein ☐		
CAD5a	(Filterfrage, falls „Ja“)<br>Empfinden Sie dies als belastend?	In sehr hohem Maße	in hohem Maße	zum Teil	in geringem Maße	in sehr geringem Maße	gar nicht
		☐	☐	☐	☐	☐	☐
CAD6	Der Distanzunterricht wurde durch unzureichende technische Ausstattung erschwert (z. B. nicht vorhandene oder veraltete Geräte).	Ja ☐			Nein ☐		
CAD6a	(Filterfrage, falls „Ja“)<br>Empfinden Sie dies als belastend?	In sehr hohem Maße	in hohem Maße	zum Teil	in geringem Maße	in sehr geringem Maße	gar nicht
		☐	☐	☐	☐	☐	☐
CAD6b	(Filterfrage Falls CAD6 „zum Teil“ ODER „in hohem Maße“ ODER „in sehr hohem Maße“)<br>Durch wessen technische Ausstattung wurde die Umstellung auf Distanzunterricht erschwert?	☐ Technische Ausstattung der Schüler*innen	☐ Schule	☐ Eigene technische Ausstattung			

	(Mehrfachnennungen möglich)						
CAD7	Der Distanzunterricht brachte mit sich, dass Sie sich häufig von den Aufgaben überfordert fühlten.	Ja ☐			Nein ☐		
CAD7a	(Filterfrage, falls „Ja“)<br>Empfinden Sie dies als belastend?	In sehr hohem Maße	in hohem Maße	zum Teil	in geringem Maße	in sehr geringem Maße	gar nicht
		☐	☐	☐	☐	☐	☐
CAD8	Der Distanzunterricht erschwerte die Leistungsbewertung Ihrer Schüler*innen.	Ja ☐			Nein ☐		
CAD8a	(Filterfrage, falls „Ja“)<br>Empfinden Sie dies als belastend?	In sehr hohem Maße	in hohem Maße	zum Teil	in geringem Maße	in sehr geringem Maße	gar nicht
		☐	☐	☐	☐	☐	☐

Einstellungen zu digitalen Unterrichtsformaten

2d)							
		Täglich	Mehrmals in der Woche	Etwa einmal in der Woche	Etwa einmal im Monat	Seltener als einmal im Monat	Fast nie / nie
CAD9	Wie oft haben Sie **vor Beginn der COVID-19-Pandemie** digitale Unterrichtsformate eingesetzt? (Einsatz von Laptops / Tablets, Online-Lernplattformen, Bereitstellung von Downloads etc.)	☐	☐	☐	☐	☐	☐
	**Die Nutzung digitaler Unterrichtsformate…**	Stimme überhaupt nicht zu	Stimme eher nicht zu	Teils / teils	Stimme eher zu	Stimme voll und ganz zu	Keine Antwort
CAD10	… bewerte ich insgesamt positiv.	☐	☐	☐	☐	☐	☐
CAD11	… hatte positive Auswirkungen auf den Leistungsstand meiner Schüler*innen.	☐	☐	☐	☐	☐	☐
CAD12	… verstärkte soziale Ungleichheit zwischen Schüler*innen (z. B. aufgrund unterschiedlicher Unterstützungsmöglichkeiten durch deren Erziehungsberechtigte).	☐	☐	☐	☐	☐	☐
CAD13	… stellt eine Chance dar.	☐	☐	☐	☐	☐	☐
CAD13a	Falls **CAD13** teils / teils oder darüber:<br><br>Für wen stellt die Umstellung auf digitale Unterrichtsformate eine Chance dar?<br><br>(Mehrfachnennungen möglich)	☐ Schüler*innen	☐ Familien der Schüler*innen	☐ Schulen	☐ Lehrkräfte	☐ Gesellschaft insgesamt	

2d)							
		Stimme überhaupt nicht zu	Stimme eher nicht zu	Teils / teils	Stimme eher zu	Stimme voll und ganz zu	Keine Antwort
CAD14	An Ihrer Schule / Dienststelle liegt ein einheitliches Gesamtkonzept zur Durchführung digitalen Unterrichts vor.	☐	☐	☐	☐	☐	☐
CAD15	Sie haben Schüler*innen Rückmeldungen zu deren Lernergebnissen aus digitalen Unterrichtsformaten gegeben.	☐	☐	☐	☐	☐	☐
BEL	Alles in allem, welche noch nicht erfragten Belastungen oder Herausforderungen stehen für Sie in Zusammenhang mit der COVID-19-Pandemie?<br><br>*(Bitte formulieren Sie Ihre Antwort knapp / in Stichworten)*	[Freitext]					

3. Umsetzung, Kommunikation und Einhaltung von Hygieneplänen

Die nachfolgenden Aussagen und Fragen beziehen sich auf die Zeit seit dem Ausbruch der COVID-19-Pandemie sowie die damit verbundenen Maßnahmen zum Infektionsschutz in Deutschland, also den Zeitraum seit etwa März 2020.

3a) persönlich

Im folgenden Abschnitt geht es um allgemeine persönliche Belastungen und Herausforderungen, die sich während der COVID-19-Pandemie ergeben haben können.

Bitte beantworten Sie die Fragen spontan. Es gibt keine „falschen Antworten“, was immer Ihnen zutreffend erscheint, ist richtig.

3a)						
		Absolut sinnlos und unangemessen	Überwiegend sinnlos und unangemessen	Weder noch	Überwiegend sinnvoll und angemessen	Absolut sinnvoll und angemessen
IFP1	Wie bewerten Sie die getroffenen Maßnahmen („AHA-Regeln“) zur Eindämmung des SARS-CoV-2-Virus?	☐	☐	☐	☐	☐
IFP2	Wie schätzen Sie das Tragen einer Mund-Nase-Bedeckung ein?	☐	☐	☐	☐	☐
IFP3	Wie bewerten Sie die Vorgabe zur Einhaltung von Abstandsregeln?	☐	☐	☐	☐	☐
IFP4	Wie bewerten Sie frühzeitige Schulschließungen im Falle steigender COVID-19-Fallzahlen als Maßnahme?	☐	☐	☐	☐	☐

3a)			
IFP5	Haben Sie die Corona-Warn-App installiert?	☐ ja	☐ nein

Intensität Gesundheitsbezogenes Informationsverhalten

IFP6	Wenn Sie einmal an eine ganz gewöhnliche Woche während der COVID-19-Pandemie denken: An wie vielen Tagen dieser Woche informieren Sie sich über das Thema COVID-19?	*An [Freitext] Tagen*

Gesundheitsbezogene Informationsquellen

IFP7	**Was sind für Sie die relevantesten Informationsquellen, aus denen Sie verbindliche Informationen rund um das Thema COVID-19-Pandemie beziehen?**<br><br>(Mehrfachnennung möglich)
☐	in persönlichen Gesprächen mit Ärzt*innen, Therapeut*innen, Pflegekräften
☐	in persönlichen Gesprächen mit Apotheker*innen
☐	in persönlichen Gesprächen mit Familienangehörigen, Freund*innen, Kolleg*innen
☐	in persönlichen Gesprächen mit anderen Patient*innen oder Betroffenen (z. B. in einer Selbsthilfegruppe, im Wartezimmer)
☐	in Beratungsstellen, Gesundheits- oder Bildungseinrichtungen
☐	am Telefon bei Krankenkassen, Patienten- oder Verbraucherschutzorganisationen
☐	in kostenlosen Broschüren oder Zeitschriften von Krankenkassen, Apotheken oder anderen Anbietern (Printausgaben; offline)
☐	in Büchern, Gesundheitsratgebern, Lexika
☐	in Zeitungen oder Zeitschriften (z. B. Printausgabe der Tageszeitung; offline)
☐	im Radio (z. B. Auto- oder Küchenradio; offline)
☐	im Fernsehen (z. B. Kabel- oder Satelliten-Fernsehen; offline)

☐	auf sonstigem Wege (offline)
☐	Wikipedia oder andere Online-Lexika
☐	Internetseiten von Krankenkassen
☐	Gesundheitsportale (z. B. netdoktor, onmeda, gesundheit.de)
☐	Webseiten von Ärzt*innen, Krankenhäusern, Reha- oder Pflegeeinrichtungen
☐	Internetauftritte von Fachstellen (z. B. RKI, BAuA, UBA usw.)
☐	Ratgeber-Communitys (z. B. gutefrage.de, wer-weiss-was.de)
☐	Online- / Internetapotheken
☐	Gesundheitsforen und Communitys speziell zu Gesundheits- und Krankheitsthemen
☐	Vergleichsportale zur Suche von Ärzt*innen, Krankenhäusern und Pflegeheimen (z. B. Weiße Liste)
☐	Social Media (z. B. Facebook, Instagram, Snapchat, Twitter usw.)
☐	Blogs zu Gesundheitsthemen
☐	Webseiten gemeinnütziger Gesundheitsorganisationen, unabhängiger Patienten- oder Selbsthilfeorganisationen
☐	Medizinische Online-Beratung (z. B. Online-Sprechstunden von Ärzt*innen oder Krankenhäusern)
☐	Online-Nachrichtenseiten (z. B. tagesschau.de, Spiegel.de, bild.de, Zeit.de, faz.net)
☐	Videoplattformen (z. B. YouTube)
☐	Online-Radio, Audio-Streaming & Podcasts
☐	Online-TV & Video-Streaming (z. B. Netflix, Amazon Prime Video usw.)

☐	Suchmaschinen (z. B. Google)
☐	Sonstige Online-Angebote

3b) Schulspezifisch

Im folgenden Abschnitt geht es um die Belastungen und Herausforderungen, die sich für Sie durch die Umsetzung, Kommunikation und Einhaltung von Hygieneplänen im Schulbetrieb während der COVID-19-Pandemie ergeben haben können.

Bitte beantworten Sie die Fragen spontan. Es gibt keine „falschen Antworten“, was immer Ihnen zutreffend erscheint, ist richtig.

3b							
		Stimme überhaupt nicht zu	Stimme eher nicht zu	Teils / teils	Stimme eher zu	Stimme voll und ganz zu	Keine Antwort
CHP1	Durch die **Hygienepläne der für Ihre Schule / Dienststelle zuständigen Landesbehörde** zum Infektionsschutz an Schulen fühlen Sie sich gut geschützt.	☐	☐	☐	☐	☐	☐
CHP2	Sie sind mit der **Umsetzung der Hygienepläne durch die Schulleitung** an Ihrer Schule zufrieden.	☐	☐	☐	☐	☐	☐
CHP3	Sie fühlen sich über Hygienepläne zum Infektionsschutz an Ihrer Schule / Dienststelle gut informiert.	☐	☐	☐	☐	☐	☐
CHP4	Schüler*innen und deren Erziehungsberechtigte sind gut über die jeweils gültigen Hygienepläne informiert.	☐	☐	☐	☐	☐	☐

3b									
		< 1 Jahr	1 – 2 Jahre	2 – 3 Jahre	3 – 5 Jahre	5 – 10 Jahre	> 10 Jahre	noch nie teilgenommen	ich weiß nicht
CHP5	Wie lange liegt Ihre letzte Teilnahme an einer Infektionsschutzbelehrung zurück?	☐	☐	☐	☐	☐	☐	☐	☐

Der Arbeitgeber hat durch eine Beurteilung der für die Beschäftigten mit ihrer Arbeit verbundenen Gefährdung zu ermitteln, welche Maßnahmen des Arbeitsschutzes erforderlich sind (§5 ArbSchG).

CHP6	Wurde an Ihrer Schule bereits eine Gefährdungsbeurteilung durchgeführt?	Ja ☐	Nein ☐	ich weiß nicht ☐
CHP7	Wurde eine Gefährdungsbeurteilung im Rahmen der COVID-19-Pandemie aktualisiert?	Ja ☐	Nein ☐	ich weiß nicht ☐

Der Arbeitgeber hat die Beschäftigten über Sicherheit und Gesundheitsschutz bei der Arbeit während ihrer Arbeitszeit ausreichend und angemessen zu unterweisen (§12 ArbSchG). Die Wahl des Mediums für die Unterweisung ist dem Arbeitgeber freigestellt.

CHP8	Fanden aufgrund der COVID-19-Pandemie Unterweisungen zu den besonderen Gefährdungen statt?	Ja ☐	Nein ☐	ich weiß nicht ☐
CHP9	Fanden aufgrund der COVID-19-Pandemie Unterweisungen zum Arbeiten im Home-Office statt?	Ja ☐	Nein ☐	ich weiß nicht ☐
CHP10	Fanden aufgrund der COVID-19-Pandemie Unterweisungen zum Schutz besonders gefährdeter Personen statt?	Ja ☐	Nein ☐	ich weiß nicht ☐
CHP11	Wurden Sie von Ihrem Dienstherren über den korrekten Gebrauch von Mund-Nase-Bedeckungen informiert?	Ja ☐	Nein ☐	ich weiß nicht ☐

3b				
CHP12	Wurden in Ihrem Schulalltag Zeiten realisiert, in denen keine Mund-Nase-Bedeckung getragen wurde (sog. „Maskenpausen“)?	Ja<br>☐	Nein<br>☐	ich weiß nicht<br>☐

3b)							
		Stimme überhaupt nicht zu	Stimme eher nicht zu	Teils / teils	Stimme eher zu	Stimme voll und ganz zu	Keine Antwort
CHC1	An Ihrer Schule / Dienststelle werden die vorgeschriebenen Hygienepläne zum Umgang mit der COVID-19-Pandemie insgesamt eingehalten.	☐	☐	☐	☐	☐	☐
	Die vorgeschriebenen Hygienepläne werden eingehalten von...						
CHC2	… Schüler*innen.	☐	☐	☐	☐	☐	☐
CHC3	… Erziehungsberechtigten der Schüler*innen.	☐	☐	☐	☐	☐	☐
CHC4	… Lehrkräften.	☐	☐	☐	☐	☐	☐
CHC5	… Hausmeister / Reinigungskräften.	☐	☐	☐	☐	☐	☐
CHC6	… sonstigen Personen.	☐	☐	☐	☐	☐	☐

3b)								
CHC7	Kam es an Ihrer Schule zu Personalausfällen aufgrund von COVID-19 (z. B. wegen Zugehörigkeit zu Risikogruppen)?	Ja ☐			Nein ☐		
CHC7a	(Filterfrage, falls „Ja“)<br>Empfinden Sie diese Veränderung als belastend?	in sehr hohem Maße	in hohem Maße	zum Teil	in geringem Maße	in sehr geringem Maße	gar nicht
		☐	☐	☐	☐	☐	☐

3b)							
ABC1	Eine Stoß- bzw. Querlüftung oder der Betrieb vorhandener raumlufttechnischer Anlagen ist möglich …	In keinem Fall	Stimme eher nicht zu	Teils / teils	Stimme eher zu	Stimme voll und ganz zu	Keine Antwort
ABC1a	… in Klassenräumen.	☐	☐	☐	☐	☐	☐
ABC1b	… auf Fluren.	☐	☐	☐	☐	☐	☐
ABC1c	… im Lehrerzimmer.	☐	☐	☐	☐	☐	☐
ABC1d	… in sanitären Einrichtungen.	☐	☐	☐	☐	☐	☐
ABC1e	… in der Mensa / im Speisesaal.	☐	☐	☐	☐	☐	☐
ABC1f	… in Sportstätten.	☐	☐	☐	☐	☐	☐
ABC1g	… in sonstigen Bereichen	☐	☐	☐	☐	☐	☐

3b)					
ABC2	Die Vorgaben zum infektionsschutzgerechten Lüften werden umgesetzt mit Hilfe von…<br><br>*(Mehrfachnennung möglich)*	☐<br>… raumlufttechnischen Anlagen	☐<br>… Fenstern mit Dauerlüften	☐<br>… Fenstern mit Stoßlüften	☐<br>… sonstigen Maßnahmen

ABC3	Es ist möglich den vorgesehenen Mindestabstand einzuhalten…						
ABC3a	… in Klassenräumen.	☐	☐	☐	☐	☐	☐
ABC3b	… auf Fluren.	☐	☐	☐	☐	☐	☐
ABC3c	… im Lehrerzimmer.	☐	☐	☐	☐	☐	☐
ABC3d	… in sanitären Einrichtungen.	☐	☐	☐	☐	☐	☐
ABC3e	… auf dem Schulhof.	☐	☐	☐	☐	☐	☐
ABC3f	… in der Mensa / im Speisesaal.	☐	☐	☐	☐	☐	☐
ABC3g	… in Sportstätten.						
ABC3h	… auf dem Schulweg.	☐	☐	☐	☐	☐	☐
ABC3i	… in sonstigen Bereichen.	☐	☐	☐	☐	☐	☐

3b)								
ABC40	Welche Art von Maske tragen Sie im Schulalltag?<br><br>*(Mehrfachnennung möglich)*	☐ Alltagsmaske (aus Stoff)		☐ Einweg-OP-Maske		☐ FFP2-Maske	☐ FFP3-Maske	
ABC4	Das korrekte Tragen einer Mund-Nase-Bedeckung (über Mund und Nase) wird umgesetzt ...							
ABC4a	... in Klassenräumen (außerhalb des Unterrichts).	☐	☐	☐	☐	☐	☐	
ABC4b	... in Klassenräumen (während des Unterrichts).	☐	☐	☐	☐	☐	☐	
ABC4c	... auf Fluren.	☐	☐	☐	☐	☐	☐	
ABC4d	... im Lehrerzimmer.	☐	☐	☐	☐	☐	☐	
ABC4e	... in sanitären Einrichtungen.	☐	☐	☐	☐	☐	☐	
ABC4f	... auf dem Schulhof.	☐	☐	☐	☐	☐	☐	
ABC4g	... in der Mensa / im Speisesaal.	☐	☐	☐	☐	☐	☐	
ABC4h	... in Sportstätten.	☐	☐	☐	☐	☐	☐	
ABC4i	... auf dem Schulweg.	☐	☐	☐	☐	☐	☐	
ABC4j	... in sonstigen Bereichen.	☐	☐	☐	☐	☐	☐	

3b)							
ABC5	An Ihrer Schule / Dienststelle ist es möglich eine hygienische Händereinigung durchzuführen (Händewaschen mit Seife und Einmalhandtüchern oder Händedesinfektion).	☐	☐	☐	☐	☐	☐

4. Auswirkungen des Schulbetriebs während der COVID-19-Pandemie auf Schulbedienstete

Die nachfolgenden Aussagen und Fragen beziehen sich auf die Zeit seit dem Ausbruch der COVID-19-Pandemie sowie die damit verbundenen Maßnahmen zum Infektionsschutz in Deutschland, also den Zeitraum seit etwa März 2020.

4a) Arbeitsbezogene Auswirkungen

Im folgenden Abschnitt geht es um die Bewertung Ihrer Arbeitstätigkeit sowie diesbezügliche Auswirkungen der COVID-19-Pandemie (denken Sie dabei bitte sowohl Phasen des Distanzunterrichts als auch Phasen des Präsenzunterrichts).

Bitte beantworten Sie die Fragen spontan. Es gibt keine „falschen Antworten“, was immer Ihnen zutreffend erscheint, ist richtig.

4a)						
		überhaupt nicht	wenig	ziemlich	sehr stark	extrem
AZ1	Wie zufrieden sind Sie mit Ihrer beruflichen Situation insgesamt?	☐	☐	☐	☐	☐
		Derzeit viel besser als vor der COVID-19-Pandemie	Derzeit etwas besser als vor der COVID-19-Pandemie	Etwa so wie vor der COVID-19-Pandemie	Derzeit etwas schlechter als vor der COVID-19-Pandemie	Derzeit viel schlechter als vor der COVID-19-Pandemie
AZ1a	Wie würden Sie diesen Aspekt im Vergleich zu vor der COVID-19-Pandemie beschreiben?	☐	☐	☐	☐	☐

4a)						
		immer	oft	manchmal	selten	nie / fast nie
AZ2	Wie oft kommt es vor, dass Sie nicht genügend Zeit haben, alle Ihre Aufgaben zu erledigen?	☐	☐	☐	☐	☐
		Derzeit viel häufiger als vor der COVID-19-Pandemie	Derzeit etwas häufiger als vor der COVID-19-Pandemie	Etwa so häufig wie vor der COVID-19-Pandemie	Derzeit etwas seltener als vor der COVID-19-Pandemie	Derzeit viel seltener als vor der COVID-19-Pandemie
AZ2a	Wie würden Sie diesen Aspekt im Vergleich zu vor der COVID-19-Pandemie beschreiben?	☐	☐	☐	☐	☐

4a)						
		in sehr hohem Maße	in hohem Maße	zum Teil	in geringem Maße	in sehr geringem Maße
SI1	Ist Ihre Arbeit sinnvoll?	☐	☐	☐	☐	☐
		Derzeit viel mehr als vor der COVID-19-Pandemie	Derzeit etwas mehr als vor der COVID-19-Pandemie	Etwa so wie vor der COVID-19-Pandemie	Derzeit etwas weniger als vor der COVID-19-Pandemie	Derzeit viel weniger als vor der COVID-19-Pandemie
SI1a	Wie würden Sie diesen Aspekt im Vergleich zu vor der COVID-19-Pandemie beschreiben?	☐	☐	☐	☐	☐

4a)						
		in sehr hohem Maße	in hohem Maße	zum Teil	in geringem Maße	in sehr geringem Maße
PR1	Werden Sie rechtzeitig im Voraus über Veränderungen an Ihrem Arbeitsplatz informiert, z.B. über wichtige Entscheidungen, Veränderungen oder Pläne für die Zukunft?	☐	☐	☐	☐	☐
		Derzeit viel mehr als vor der COVID-19-Pandemie	Derzeit etwas mehr als vor der COVID-19-Pandemie	Etwa so wie vor der COVID-19-Pandemie	Derzeit etwas weniger als vor der COVID-19-Pandemie	Derzeit viel weniger als vor der COVID-19-Pandemie
PR1a	Wie würden Sie diesen Aspekt im Vergleich zu vor der COVID-19-Pandemie beschreiben?	☐	☐	☐	☐	☐
		in sehr hohem Maße	in hohem Maße	zum Teil	in geringem Maße	in sehr geringem Maße
PR2	Erhalten Sie alle Informationen, die Sie brauchen, um Ihre Arbeit gut zu erledigen?	☐	☐	☐	☐	☐
		Derzeit viel mehr als vor der COVID-19-Pandemie	Derzeit etwas mehr als vor der COVID-19-Pandemie	Etwa so wie vor der COVID-19-Pandemie	Derzeit etwas weniger als vor der COVID-19-Pandemie	Derzeit viel weniger als vor der COVID-19-Pandemie
PR2a	Wie würden Sie diesen Aspekt im Vergleich zu vor der COVID-19-Pandemie beschreiben?	☐	☐	☐	☐	☐

4a)						
		immer	oft	manchmal	selten	nie / fast nie
EIN1	Haben Sie großen Einfluss auf Entscheidungen, die Ihre Arbeit betreffen?	☐	☐	☐	☐	☐
		Derzeit viel mehr als vor der COVID-19-Pandemie	Derzeit etwas mehr als vor der COVID-19-Pandemie	Etwa so wie vor der COVID-19-Pandemie	Derzeit etwas weniger als vor der COVID-19-Pandemie	Derzeit viel weniger als vor der COVID-19-Pandemie
EIN1a	Wie würden Sie diesen Aspekt im Vergleich zu vor der COVID-19-Pandemie beschreiben?	☐	☐	☐	☐	☐
		immer	oft	manchmal	selten	nie / fast nie
EIN2	Haben Sie Einfluss auf die Menge der Arbeit, die Ihnen übertragen wird?	☐	☐	☐	☐	☐
		Derzeit viel mehr als vor der COVID-19-Pandemie	Derzeit etwas mehr als vor der COVID-19-Pandemie	Etwa so wie vor der COVID-19-Pandemie	Derzeit etwas weniger als vor der COVID-19-Pandemie	Derzeit viel weniger als vor der COVID-19-Pandemie
EIN2a	Wie würden Sie diesen Aspekt im Vergleich zu vor der COVID-19-Pandemie beschreiben?	☐	☐	☐	☐	☐

4a)						
		immer	oft	manchmal	selten	nie / fast nie
EIN3	Haben Sie Einfluss darauf, was Sie bei Ihrer Arbeit tun?	☐	☐	☐	☐	☐
		Derzeit viel mehr als vor der COVID-19-Pandemie	Derzeit etwas mehr als vor der COVID-19-Pandemie	Etwa so wie vor der COVID-19-Pandemie	Derzeit etwas weniger als vor der COVID-19-Pandemie	Derzeit viel weniger als vor der COVID-19-Pandemie
EIN3a	Wie würden Sie diesen Aspekt im Vergleich zu vor der COVID-19-Pandemie beschreiben?	☐	☐	☐	☐	☐

4a)						
		immer	oft	manchmal	selten	nie / fast nie
EMO1	Gehört es zu Ihrer Arbeit, sich mit den persönlichen Problemen anderer Menschen zu beschäftigen?	☐	☐	☐	☐	☐
		Derzeit viel mehr als vor der COVID-19-Pandemie	Derzeit etwas mehr als vor der COVID-19-Pandemie	Etwa so wie vor der COVID-19-Pandemie	Derzeit etwas weniger als vor der COVID-19-Pandemie	Derzeit viel weniger als vor der COVID-19-Pandemie
EMO1a	Wie würden Sie diesen Aspekt im Vergleich zu vor der COVID-19-Pandemie beschreiben?	☐	☐	☐	☐	☐

4a)						
		in sehr hohem Maße	in hohem Maße	zum Teil	in geringem Maße	in sehr geringem Maße
EMO2	Ist Ihre Arbeit emotional fordernd?	☐	☐	☐	☐	☐
		Derzeit viel mehr als vor der COVID-19-Pandemie	Derzeit etwas mehr als vor der COVID-19-Pandemie	Etwa so wie vor der COVID-19-Pandemie	Derzeit etwas weniger als vor der COVID-19-Pandemie	Derzeit viel weniger als vor der COVID-19-Pandemie
EMO2a	Wie würden Sie diesen Aspekt im Vergleich zu vor der COVID-19-Pandemie beschreiben?	☐	☐	☐	☐	☐

4a)						
		in sehr hohem Maße	in hohem Maße	zum Teil	in geringem Maße	in sehr geringem Maße
VER1	Verlangt Ihre Arbeit von Ihnen, dass Sie Ihre Gefühle verbergen?	☐	☐	☐	☐	☐
		Derzeit viel mehr als vor der COVID-19-Pandemie	Derzeit etwas mehr als vor der COVID-19-Pandemie	Etwa so wie vor der COVID-19-Pandemie	Derzeit etwas weniger als vor der COVID-19-Pandemie	Derzeit viel weniger als vor der COVID-19-Pandemie
VER1a	Wie würden Sie diesen Aspekt im Vergleich zu vor der COVID-19-Pandemie beschreiben?	☐	☐	☐	☐	☐

4a)						
		in sehr hohem Maße	in hohem Maße	zum Teil	in geringem Maße	in sehr geringem Maße
VER2	Verlangt Ihre Arbeit von Ihnen, sich mit Ihrer Meinung zurück zu halten?	☐	☐	☐	☐	☐
		Derzeit viel mehr als vor der COVID-19-Pandemie	Derzeit etwas mehr als vor der COVID-19-Pandemie	Etwa so wie vor der COVID-19-Pandemie	Derzeit etwas weniger als vor der COVID-19-Pandemie	Derzeit viel weniger als vor der COVID-19-Pandemie
VER2a	Wie würden Sie diesen Aspekt im Vergleich zu vor der COVID-19-Pandemie beschreiben?	☐	☐	☐	☐	☐

4a)						
		in sehr hohem Maße	in hohem Maße	zum Teil	in geringem Maße	in sehr geringem Maße
WPK3	Meine Arbeit beansprucht so viel Energie, dass sich dies negativ auf mein Privatleben auswirkt.	☐	☐	☐	☐	☐
		Derzeit viel mehr als vor der COVID-19-Pandemie	Derzeit etwas mehr als vor der COVID-19-Pandemie	Etwa so wie vor der COVID-19-Pandemie	Derzeit etwas weniger als vor der COVID-19-Pandemie	Derzeit viel weniger als vor der COVID-19-Pandemie
WPK3a	Wie würden Sie diesen Aspekt im Vergleich zu vor der COVID-19-Pandemie beschreiben?	☐	☐	☐	☐	☐

4a)						
		in sehr hohem Maße	in hohem Maße	zum Teil	in geringem Maße	in sehr geringem Maße
WPK4	Meine Arbeit nimmt so viel Zeit in Anspruch, dass sich dies negativ auf mein Privatleben auswirkt.	☐	☐	☐	☐	☐
		Derzeit viel mehr als vor der COVID-19-Pandemie	Derzeit etwas mehr als vor der COVID-19-Pandemie	Etwa so wie vor der COVID-19-Pandemie	Derzeit etwas weniger als vor der COVID-19-Pandemie	Derzeit viel weniger als vor der COVID-19-Pandemie
WPK4a	Wie würden Sie diesen Aspekt im Vergleich zu vor der COVID-19-Pandemie beschreiben?	☐	☐	☐	☐	☐

4a)						
		immer	oft	manchmal	selten	nie / fast nie
UNT1	Wie oft erhalten Sie bei Bedarf Hilfe und Unterstützung von Ihren Kollegen/innen?	☐	☐	☐	☐	☐
		Derzeit viel mehr als vor der COVID-19-Pandemie	Derzeit etwas mehr als vor der COVID-19-Pandemie	Etwa so wie vor der COVID-19-Pandemie	Derzeit etwas weniger als vor der COVID-19-Pandemie	Derzeit viel weniger als vor der COVID-19-Pandemie
UNT1a	Wie würden Sie diesen Aspekt im Vergleich zu vor der COVID-19-Pandemie beschreiben?	☐	☐	☐	☐	☐

4a)						
		immer	oft	manchmal	selten	nie / fast nie
UNT2	Wie oft erhalten Sie bei Bedarf Hilfe und Unterstützung von Ihrem/Ihrer unmittelbaren Vorgesetzten?	☐	☐	☐	☐	☐
		Derzeit viel häufiger als vor der COVID-19-Pandemie	Derzeit etwas häufiger als vor der COVID-19-Pandemie	Etwa so häufig wie vor der COVID-19-Pandemie	Derzeit etwas seltener als vor der COVID-19-Pandemie	Derzeit viel seltener als vor der COVID-19-Pandemie
UNT2a	Wie würden Sie diesen Aspekt im Vergleich zu vor der COVID-19-Pandemie beschreiben?	☐	☐	☐	☐	☐

4a)						
		immer	oft	manchmal	selten	nie / fast nie
FB1	Wie oft spricht Ihr/e Vorgesetzte/r mit Ihnen über die Qualität Ihrer Arbeit?	☐	☐	☐	☐	☐
		Derzeit viel häufiger als vor der COVID-19-Pandemie	Derzeit etwas häufiger als vor der COVID-19-Pandemie	Etwa so häufig wie vor der COVID-19-Pandemie	Derzeit etwas seltener als vor der COVID-19-Pandemie	Derzeit viel seltener als vor der COVID-19-Pandemie
FB1a	Wie würden Sie diesen Aspekt im Vergleich zu vor der COVID-19-Pandemie beschreiben?	☐	☐	☐	☐	☐

4a)						
		immer	oft	manchmal	selten	nie / fast nie
FB2	Wie oft sprechen Ihre Kollegen/innen mit Ihnen über die Qualität Ihrer Arbeit?	☐	☐	☐	☐	☐
		Derzeit viel häufiger als vor der COVID-19-Pandemie	Derzeit etwas häufiger als vor der COVID-19-Pandemie	Etwa so häufig wie vor der COVID-19-Pandemie	Derzeit etwas seltener als vor der COVID-19-Pandemie	Derzeit viel seltener als vor der COVID-19-Pandemie
FB2a	Wie würden Sie diesen Aspekt im Vergleich zu vor der COVID-19-Pandemie beschreiben?	☐	☐	☐	☐	☐

4a)						
		immer	oft	manchmal	selten	nie / fast nie
UNG	Wie oft fühlen Sie sich durch Kollegen/innen oder Vorgesetzte zu Unrecht kritisiert, schikaniert oder vor anderen bloßgestellt?	☐	☐	☐	☐	☐
		Derzeit viel häufiger als vor der COVID-19-Pandemie	Derzeit etwas häufiger als vor der COVID-19-Pandemie	Etwa so häufig wie vor der COVID-19-Pandemie	Derzeit etwas seltener als vor der COVID-19-Pandemie	Derzeit viel seltener als vor der COVID-19-Pandemie
UNGa	Wie würden Sie diesen Aspekt im Vergleich zu vor der COVID-19-Pandemie beschreiben?	☐	☐	☐	☐	☐

4a)

**Bitte überprüfen Sie, wie sehr Sie das folgende Problem <u>während der letzten Woche</u> belastet hat.**		Überhaupt nicht	Wenig	Ziemlich	Sehr stark	Extrem
IFL1	Wie stark fühlen Sie sich durch Ihre berufliche Situation belastet?	☐	☐	☐	☐	☐
		Derzeit viel mehr als vor der COVID-19-Pandemie	Derzeit etwas mehr als vor der COVID-19-Pandemie	Etwa so wie vor der COVID-19-Pandemie	Derzeit etwas weniger als vor der COVID-19-Pandemie	Derzeit viel weniger als vor der COVID-19-Pandemie
IFL1a	Wie würden Sie diesen Aspekt im Vergleich zu vor der COVID-19-Pandemie beschreiben?	☐	☐	☐	☐	☐

**Bitte überprüfen Sie, wie sehr Sie das folgende Problem <u>während der letzten Woche</u> belastet hat.**		Starke Ablehnung	Ablehnung	Leichte Ablehnung	Leichte Zustimmung	Zustimmung	Starke Zustimmung
IFL2	Im Berufsalltag habe ich oftmals das Gefühl, dass ich ungerecht behandelt werde.	☐	☐	☐	☐	☐	☐

		Derzeit viel mehr als vor der COVID-19-Pandemie	Derzeit etwas mehr als vor der COVID-19-Pandemie	Etwa so wie vor der COVID-19-Pandemie	Derzeit etwas weniger als vor der COVID-19-Pandemie	Derzeit viel weniger als vor der COVID-19-Pandemie
IFL2a	Wie würden Sie diesen Aspekt im Vergleich zu vor der COVID-19-Pandemie beschreiben?	☐	☐	☐	☐	☐

4a)						
**Bitte überprüfen Sie, wie sehr Sie das folgende Problem <u>während der letzten Woche</u> belastet hat.**		Sicher	Eher ja	Unsicher	Eher nein	Auf keinen Fall
IFL3	Wenn Sie an Ihren Gesundheitszustand und Ihre berufliche Leistungsfähigkeit denken: Glauben Sie, dass Sie bis zum Erreichen des Pensionsalters berufstätig sein können?	☐	☐	☐	☐	☐
		Derzeit viel mehr als vor der COVID-19-Pandemie	Derzeit etwas mehr als vor der COVID-19-Pandemie	Etwa so wie vor der COVID-19-Pandemie	Derzeit etwas weniger als vor der COVID-19-Pandemie	Derzeit viel weniger als vor der COVID-19-Pandemie
IFL3a	Wie würden Sie diesen Aspekt im Vergleich zu vor der COVID-19-Pandemie beschreiben?	☐	☐	☐	☐	☐

4b) Beanspruchungen (somatisch / psychisch) sowie private / soziale Herausforderungen

Im folgenden Abschnitt geht es um körperliche, psychische sowie soziale Aspekte.

Bitte beantworten Sie die Fragen spontan. Es gibt keine „falschen Antworten“, was immer Ihnen zutreffend erscheint, ist richtig.

4b) WHO- self rated general health							
		sehr schlecht	schlecht	nicht ganz zufriedenstellend	zufriedenstellend	gut	sehr gut
WHO1	Wie würden Sie Ihren Gesundheitszustand im Allgemeinen beschreiben?	☐	☐	☐	☐	☐	☐
		Derzeit viel besser als vor der COVID-19-Pandemie	Derzeit etwas besser als vor der COVID-19-Pandemie	Etwa so wie vor der COVID-19-Pandemie	Derzeit etwas schlechter als vor der COVID-19-Pandemie	Derzeit viel schlechter als vor der COVID-19-Pandemie	
WHO1a	Wie würden Sie diesen Aspekt im Vergleich zu vor der COVID-19-Pandemie beschreiben?	☐	☐	☐	☐	☐	

**4b)**			
COV1	Mussten Sie sich aufgrund eines Verdachtes auf COVID-19 (z. B. Symptome, Kontakt zu Personen mit bestätigter Erkrankung, Rückkehr aus Risikogebiet) in häusliche Quarantäne begeben?	☐ ja	☐ nein

COV2	Sind Sie aktuell oder waren Sie in der Vergangenheit bereits mit dem SARS-CoV-2-Virus infiziert (COVID-19)?	☐ ja			☐ nein	
COV2a	(Filterfrage: falls COV2 = ja)<br>Wie schwer war der Verlauf ihrer COVID-19-Erkrankung?	Keine Symptome ☐	Leicht (keine ärztliche Betreuung erforderlich) ☐	Mittelschwer (z. B. Husten, Beeinträchtigung des Geruchssinns / hausärztliche Betreuung in Anspruch genommen) ☐	Schwer (z. B. Atemnot, starke Schmerzen, / stationäre Behandlung in Krankenhaus notwendig) ☐	Sehr schwer (Intensivmedizinische Behandlung notwendig) ☐
COV3	Ist aktuell oder war in der Vergangenheit eine Ihnen nahestehende Person mit dem SARS-CoV-2-Virus infiziert (COVID-19)?	☐ ja			☐ nein	

Nachfolgend sind einige der häufigsten Symptome aufgeführt, wegen derer ärztliche Hilfe in Anspruch genommen wird.

Bitte beantworten Sie die Fragen spontan. Es gibt keine „falschen Antworten", was immer Ihnen zutreffend erscheint, ist richtig.

4b)				
	**Wie stark fühlten Sie sich im Verlauf der letzten 4 Wochen durch die folgenden Beschwerden beeinträchtigt?**	Nicht beeinträchtigt	Wenig beeinträchtigt	Stark beeinträchtigt
P1	Bauchschmerzen	☐	☐	☐
P2	Rückenschmerzen	☐	☐	☐
P3	Schmerzen in Armen, Beinen oder Gelenken (Knie, Hüften usw.)	☐	☐	☐
P4	Menstruationsschmerzen oder andere Probleme mit der Menstruation	☐	☐	☐
P5	Schmerzen oder Probleme beim Geschlechtsverkehr	☐	☐	☐
P6	Kopfschmerzen	☐	☐	☐
P7	Schmerzen im Brustbereich	☐	☐	☐
P8	Schwindel	☐	☐	☐
P9	Ohnmachtsanfälle	☐	☐	☐
P10	Herzklopfen oder Herzrasen	☐	☐	☐

4b)				
P11	Kurzatmigkeit	☐	☐	☐
P12	Verstopfung, nervöser Darm oder Durchfall	☐	☐	☐
P13	Übelkeit, Blähungen oder Verdauungsbeschwerden	☐	☐	☐

**Wie stark fühlten Sie sich im Verlauf der <u>letzten 2 Wochen</u> durch die folgenden Beschwerden beeinträchtigt?**		Überhaupt nicht	An einzelnen Tagen	An mehr als der Hälfte der Tage	Beinahe jeden Tag
P14	Schwierigkeiten, ein- oder durchzuschlafen, oder vermehrter Schlaf	☐	☐	☐	☐
P15	Müdigkeit oder Gefühl, keine Energie zu haben	☐	☐	☐	☐

Im Folgenden werden individuelle Risikofaktoren für einen schweren Krankheitsverlauf erfragt.

4b)				
**Welche der aktuell bekannten Risikofaktoren für einen schweren Krankheitsverlauf bei einer COVID-19-Erkrankung treffen auf Sie zu?**		Ja	Nein	Keine Antwort
RF1	Herz-Kreislauf-Erkrankung mit stark eingeschränkter Pumpfunktion des Herzens oder Folgeschäden (z. B. Herzinsuffizienz, koronare Herzkrankheit)	☐	☐	☐
RF2	Chronische Lungenerkrankung (z. B. COPD mit mittelmäßig bis stark eingeschränkter Lungenfunktion, Mukoviszidose)	☐	☐	☐
RF3	Schwere Magen-, Darmerkrankungen (z. B. chronisch entzündliche Darmerkrankungen mit Immunsuppression) und / oder fortgeschrittene Leberzirrhosen	☐	☐	☐
RF4	Diabetes mellitus Typ I – insulinpflichtig oder mit Folgeschäden	☐	☐	☐
RF5	Krebserkrankung unter laufender Therapie oder mit Langzeittherapiefolgen	☐	☐	☐
RF6	Immundefizit (z. B. HI-Virusinfektion, Antikörpermangelsyndrome, zu wenig weiße Blutkörperchen)	☐	☐	☐
RF7	Therapie mit Immunsuppressiva (z. B. Cortison > 5mg pro Tag, Monoklonale Antikörper, Methotrexat)	☐	☐	☐

RF8	Starkes Untergewicht (BMI <16, z.B. aufgrund von Magersucht / Anorexie)	☐	☐	☐
RF9	Lebt in Ihrem Haushalt eine Person, auf die mindestens einer der erfragten Risikofaktoren zutrifft?	☐	☐	☐
RF10	Haben Sie regelmäßig persönlichen Kontakt mit einer Ihnen nahestehenden Person (z. B. Familie, Freundeskreis, Verein), auf die mindestens einer der erfragten Risikofaktoren zutrifft?	☐	☐	☐

4b)					
Wie oft fühlten Sie sich im Verlauf der letzten 2 Wochen durch die folgenden Beschwerden beeinträchtigt?		Überhaupt nicht	An einzelnen Tagen	An mehr als der Hälfte der Tage	Beinahe jeden Tag
DEP1	Wenig Interesse oder Freude an Ihren Tätigkeiten	☐	☐	☐	☐
ANG1	Nicht in der Lage sein, Sorgen zu stoppen oder zu kontrollieren	☐	☐	☐	☐
ANG2	Nervosität, Ängstlichkeit oder Anspannung	☐	☐	☐	☐
DEP2	Niedergeschlagenheit, Schwermut oder Hoffnungslosigkeit	☐	☐	☐	☐

4b)						
		Stimme überhaupt nicht zu	Stimme eher nicht zu	Teils / teils	Stimme eher zu	Stimme voll und ganz zu
COB1	Ich fühle mich der aktuellen COVID-19-Pandemie hilflos ausgeliefert.	☐	☐	☐	☐	☐
COB2	Nicht zu wissen, wie lange die aktuelle COVID-19-Pandemie anhalten wird, bereitet mir Sorgen.	☐	☐	☐	☐	☐
COB3	Die Aussicht, während der COVID-19-Pandemie an meiner Schule / Dienststelle zu arbeiten, bereitet mir Sorgen im Hinblick auf meine Gesundheit.	☐	☐	☐	☐	☐

4b)				
CA1	Wie stark ist Ihre Angst davor, sich mit dem SARS-CoV-2-Virus zu infizieren?	0 – keine Angst	Ihr Wert	100 – sehr starke Angst
CA1a<br>Wo ist Ihre Angst sich mit dem SARS-CoV-2-Virus zu infizieren am stärksten?<br>(Mehrfachnennung möglich)		☐ Wohnstätte	☐ med. Behandlungseinrichtungen	☐ Arbeitsplatz / Schule
		☐ Betreuungseinrichtung	☐ Freizeit	☐ Speisestätte
		☐ Übernachtungsstätten	☐ Verkehrsmittel	☐ Geschäfte
		☐ Sonstiger Ort		
CA2	Wenn Sie an COVID-19 erkranken würden: Wie hoch schätzen Sie die Wahrscheinlichkeit ein, dass diese Erkrankung einen schweren Verlauf nimmt?	0 – extrem unwahrscheinlich	Ihr Wert	100 – extrem wahrscheinlich
CA3	Wie stark ist Ihre Angst davor, dass Sie selbst zum Überträger des SARS-CoV-2-Virus werden könnten, d.h. andere Personen in Ihrer Umgebung mit dem Corona-Virus anstecken könnten?	0 – keine Angst	Ihr Wert	100 – sehr starke Angst

CA4	Wie stark ist Ihre Angst davor, dass Freunde oder Angehörige sich mit dem SARS-CoV-2-Virus infizieren?	0 – keine Angst	Ihr Wert	100 – sehr starke Angst
CA5	Wie sehr beeinträchtigt Sie die Angst vor einer COVID-19-Erkrankung im Alltag?	0 – gar nicht	Ihr Wert	100 – sehr stark

4b)									
		**Nie**	**Mind. ein paar Mal im Jahr**	**Mind. einmal im Monat**	**Einige Male pro Monat**	**Einmal pro Woche**	**Mehrmals pro Woche**	**Täglich**	**Keine Antwort**
EE1	Wie oft fühlen Sie sich von Ihrer Arbeit ausgebrannt?	☐	☐	☐	☐	☐	☐	☐	☐

		Derzeit viel häufiger als vor der COVID-19-Pandemie	Derzeit etwas häufiger als vor der COVID-19-Pandemie	Etwa so häufig wie vor der COVID-19-Pandemie	Derzeit etwas seltener als vor der COVID-19-Pandemie	Derzeit viel seltener als vor der COVID-19-Pandemie
EE1a	Wie würden Sie diesen Aspekt im Vergleich zu vor der COVID-19-Pandemie beschreiben?	☐	☐	☐	☐	☐

4b)									
		**Nie**	**Mind. ein paar Mal im Jahr**	**Mind. einmal im Monat**	**Einige Male pro Monat**	**Einmal pro Woche**	**Mehrmals pro Woche**	**Täglich**	**Keine Antwort**
DP1	Wie oft haben Sie das Gefühl, dass Sie im Umgang mit anderen Menschen gefühlloser geworden sind, seit Sie diese Arbeit ausüben?	☐	☐	☐	☐	☐	☐	☐	☐

		Derzeit viel häufiger als vor der COVID-19-Pandemie	Derzeit etwas häufiger als vor der COVID-19-Pandemie	Etwa so häufig wie vor der COVID-19-Pandemie	Derzeit etwas seltener als vor der COVID-19-Pandemie	Derzeit viel seltener als vor der COVID-19-Pandemie
DP1a	Wie würden Sie diesen Aspekt im Vergleich zu vor der COVID-19-Pandemie beschreiben?	☐	☐	☐	☐	☐

Gesundheitsverhalten

4b)						
		**nie**	**1-4 Tage**	**5-9 Tage**	**10-14 Tage**	**15 Tage und mehr**
PRAE	An wie vielen Tagen ist es bei Ihnen in den letzten 12 Monaten vorgekommen, dass Sie gearbeitet haben, obwohl Sie sich richtig krank gefühlt haben?	☐	☐	☐	☐	☐

PA1	An wie vielen Tagen in der vergangenen Woche waren Sie mindestens 30 Minuten oder mehr körperlich aktiv, sodass Ihre Atemfrequenz erhöht war? Dazu kann Sport, Bewegung sowie zügiges Gehen oder Radfahren zur Erholung oder zum Erreichen von Orten gehören, jedoch nicht die Hausarbeit oder körperliche Aktivitäten, die Teil Ihrer Arbeit sind.	☐ 0 Tage	☐ 1 Tag	☐ 2 Tage	☐ 3 Tage	☐ 4 Tage	☐ 5 Tage	☐ 6 Tage	☐ 7 Tage

		Derzeit viel häufiger als vor der COVID-19-Pandemie	Derzeit etwas häufiger als vor der COVID-19-Pandemie	Etwa so häufig wie vor der COVID-19-Pandemie	Derzeit etwas seltener als vor der COVID-19-Pandemie	Derzeit viel seltener als vor der COVID-19-Pandemie
PA1a	Wie würden Sie diesen Aspekt im Vergleich zu vor der COVID-19-Pandemie beschreiben?	☐	☐	☐	☐	☐
		Stimme überhaupt nicht zu	Stimme eher nicht zu	Teils / teils	Stimme eher zu	Stimme voll und ganz zu

COB4	Ich nehme seit Beginn der COVID-19-Pandemie vermehrt Suchtmittel (z. B. Alkohol, Beruhigungsmittel) zu mir.	☐	☐	☐	☐	☐
COB5	Ich informiere mich übermäßig häufig zur aktuellen Lage der COVID-19-Pandemie.	☐	☐	☐	☐	☐

		Ja, täglich	Ja, gelegentlich	Nein, nicht mehr	Nein, ich habe noch nie geraucht
RAU	Rauchen Sie zur Zeit?	☐	☐	☐	☐

		Derzeit viel mehr als vor der COVID-19-Pandemie	Derzeit etwas mehr als vor der COVID-19-Pandemie	Etwa so wie vor der COVID-19-Pandemie	Derzeit etwas weniger als vor der COVID-19-Pandemie	Derzeit viel weniger als vor der COVID-19-Pandemie
RAUa	Wie würden Sie diesen Aspekt im Vergleich zu vor der COVID-19-Pandemie beschreiben?	☐	☐	☐	☐	☐

4b)						
		immer	oft	manchmal	selten	nie / fast nie
COPS	Wie häufig können Sie in Ihrer Freizeit die Arbeit nicht vergessen?	☐	☐	☐	☐	☐
		Derzeit viel häufiger als vor der COVID-19-Pandemie	Derzeit etwas häufiger als vor der COVID-19-Pandemie	Etwa so häufig wie vor der COVID-19-Pandemie	Derzeit etwas seltener als vor der COVID-19-Pandemie	Derzeit viel seltener als vor der COVID-19-Pandemie

4b)						
COPSa	Wie würden Sie diesen Aspekt im Vergleich zu vor der COVID-19-Pandemie beschreiben?	☐	☐	☐	☐	☐

4b)						
		trifft überhaupt nicht zu	trifft eher nicht zu	weder noch	eher zutreffend	trifft voll und ganz zu
BFI1	Ich bin eher zurückhaltend, reserviert.	☐	☐	☐	☐	☐
BFI2	Ich schenke anderen leicht Vertrauen, glaube an das Gute im Menschen.	☐	☐	☐	☐	☐
BFI3	Ich bin bequem, neige zur Faulheit.	☐	☐	☐	☐	☐
BFI4	Ich bin entspannt, lasse mich durch Stress nicht aus der Ruhe bringen.	☐	☐	☐	☐	☐
BFI5	Ich habe nur wenig künstlerisches Interesse.	☐	☐	☐	☐	☐
BFI6	Ich gehe aus mir heraus, bin gesellig.	☐	☐	☐	☐	☐
BFI7	Ich neige dazu, andere zu kritisieren.	☐	☐	☐	☐	☐
BFI8	Ich erledige Aufgaben gründlich.	☐	☐	☐	☐	☐
BFI9	Ich werde leicht nervös und unsicher.	☐	☐	☐	☐	☐

4b)						
BFI10	Ich habe eine aktive Vorstellungskraft, bin fantasievoll.	☐	☐	☐	☐	☐

4b)							
		Stimme überhaupt nicht zu	Stimme eher nicht zu	Teils / teils	Stimme eher zu	Stimme voll und ganz zu	Keine Antwort
SOZ0	Seit Beginn der COVID-19-Pandemie haben in meinem Leben <u>private</u> Belastungen insgesamt zugenommen.	☐	☐	☐	☐	☐	☐
SOZ1	In der COVID-19-Pandemie erhalte ich genügend Unterstützung (emotional, praktisch) von Personen aus meinem sozialen Umfeld.	☐	☐	☐	☐	☐	☐
SOZ2	Seit Beginn der COVID-19-Pandemie ist es in meinem Haushalt vermehrt zu Konflikten gekommen.	☐	☐	☐	☐	☐	☐

4b)							
SOZ3	Seit Beginn der COVID-19-Pandemie ist es zu Einschränkungen in meinen Freizeitaktivitäten (z. B. Sport, Vereinsarbeit, Treffen im Freundeskreis) gekommen.	☐	☐	☐	☐	☐	☐
SOZ4	Die COVID-19-Pandemie bringt mich selbst / meinen Haushalt in große wirtschaftliche Schwierigkeiten.	☐	☐	☐	☐	☐	☐
SOZ5	Mit der Betreuung meiner eigenen Kinder in Kita oder Schule, während der COVID-19-Pandemie, bin ich zufrieden.	☐	☐	☐	☐	☐	☐

		Nein<br>trifft nicht zu	Ja<br>trifft zu<br>und hat mich nicht belastet	Ja<br>trifft zu<br>und hat mich wenig belastet	Ja<br>trifft zu<br>und hat mich mittelmäßig belastet	Ja<br>trifft zu<br>und hat mich stark belastet	Keine Antwort
LLN	Ich bin häufig allein, habe wenig Kontakte.	☐	☐	☐	☐	☐	☐

		Derzeit viel häufiger als vor der COVID-19-Pandemie	Derzeit etwas häufiger als vor der COVID-19-Pandemie	Etwa so häufig wie vor der COVID-19-Pandemie	Derzeit etwas seltener als vor der COVID-19-Pandemie	Derzeit viel seltener als vor der COVID-19-Pandemie
LLNa	Wie würden Sie diesen Aspekt im Vergleich zu vor der COVID-19-Pandemie beschreiben?	☐	☐	☐	☐	☐

		Stimme überhaupt nicht zu	Stimme eher nicht zu	Teils / teils	Stimme eher zu	Stimme voll und ganz zu
RES1	Die COVID-19-Pandemie hat zu positiven Veränderungen in meinem Leben geführt.	☐	☐	☐	☐	☐

4b)						
RES2	Ich habe das Gefühl, dass ich aktiv etwas Positives für die Gesellschaft in dieser COVID-19 Pandemie tun kann (z. B. vermehrt Hilfe anbieten).	☐	☐	☐	☐	☐
RES3	Ich habe das Gefühl, dass ich aktiv etwas Positives für mich in dieser COVID-19-Pandemie tun kann.	☐	☐	☐	☐	☐

5.Sammlung von Beispielen bewährter Maßnahmen

5a) Beispiele bewährter Maßnahmen zum Infektionsschutz

Im Folgenden geht es darum genauer zu erfahren, welche COVID-19-Infektionsschutzmaßnahmen Sie an Ihrer Schule / Dienststelle als gut umsetzbar bzw. wirksam halten. Ziel ist es, die am besten bewerteten Konzepte systematisch zu erfassen, um daraus Maßnahmenempfehlungen abzuleiten.

5a)							
	**Wie schätzen Sie über unterschiedliche Situationen Ihres Schulalltags hinweg die Umsetzbarkeit sowie die Wirksamkeit folgender Infektionsschutzmaßnahmen während der COVID-19-Pandemie ein?**						
	**Tragen einer Mund-Nase-Bedeckung …**	in sehr hohem Maße	in hohem Maße	zum Teil	in geringem Maße	in sehr geringem Maße	gar nicht
BPa11	… ist umsetzbar.	☐	☐	☐	☐	☐	☐
BPa12	… halte ich für eine wirksame Infektionsschutzmaßnahme.	☐	☐	☐	☐	☐	☐
	**Abstand einhalten (min. 1,5m) …**	in sehr hohem Maße	in hohem Maße	zum Teil	in geringem Maße	in sehr geringem Maße	gar nicht
BPa21	… ist umsetzbar.	☐	☐	☐	☐	☐	☐

5a)							
BPa22	… halte ich für eine wirksame Infektionsschutzmaßnahme.	☐	☐	☐	☐	☐	☐
	**Vermeiden von Körperkontakt …**	in sehr hohem Maße	in hohem Maße	zum Teil	in geringem Maße	in sehr geringem Maße	gar nicht
BPa31	… ist umsetzbar.	☐	☐	☐	☐	☐	☐
BPa32	… halte ich für eine wirksame Infektionsschutzmaßnahme.	☐	☐	☐	☐	☐	☐
	**Befolgen von Hygienehinweisen z. B. Hinweisschilder oder Bodenmarkierungen …**	in sehr hohem Maße	in hohem Maße	zum Teil	in geringem Maße	in sehr geringem Maße	gar nicht
BPa41	… ist umsetzbar.	☐	☐	☐	☐	☐	☐
BPa42	… halte ich für eine wirksame Infektionsschutzmaßnahme.	☐	☐	☐	☐	☐	☐
	**Einhaltung eines Einbahnstraßensystems (Personen gehen nur in einer vorgeschriebenen Richtung, z. B. im Treppenhaus oder auf Fluren) …**	in sehr hohem Maße	in hohem Maße	zum Teil	in geringem Maße	in sehr geringem Maße	gar nicht
BPa51	… ist umsetzbar.	☐	☐	☐	☐	☐	☐
BPa52	… halte ich für eine wirksame Infektionsschutzmaßnahme.	☐	☐	☐	☐	☐	☐

5a)

	**Regelmäßiges Stoß-/ Querlüften …**	in sehr hohem Maße	in hohem Maße	zum Teil	in geringem Maße	in sehr geringem Maße	gar nicht
BPa611	… ist umsetzbar.	☐	☐	☐	☐	☐	☐
BPa612	… halte ich für eine wirksame Infektionsschutzmaßnahme.	☐	☐	☐	☐	☐	☐
	**Der Einsatz raumlufttechnischer Anlagen …**	in sehr hohem Maße	in hohem Maße	zum Teil	in geringem Maße	in sehr geringem Maße	gar nicht
BPa621	… ist umsetzbar.	☐	☐	☐	☐	☐	☐
BPa622	… halte ich für eine wirksame Infektionsschutzmaßnahme.	☐	☐	☐	☐	☐	☐
	**Einhalten einer festen Sitzordnung im Klassenzimmer …**	in sehr hohem Maße	in hohem Maße	zum Teil	in geringem Maße	in sehr geringem Maße	gar nicht
BPa71	… ist umsetzbar.	☐	☐	☐	☐	☐	☐
BPa72	… halte ich für eine wirksame Infektionsschutzmaßnahme.	☐	☐	☐	☐	☐	☐
	**Einhalten einer festen Sitzordnung im Lehrerzimmer …**	in sehr hohem Maße	in hohem Maße	zum Teil	in geringem Maße	in sehr geringem Maße	gar nicht
BPa81	… ist umsetzbar.	☐	☐	☐	☐	☐	☐
BPa82	… halte ich für eine wirksame Infektionsschutzmaßnahme.	☐	☐	☐	☐	☐	☐

	**Teilung / Kohortierung von Klassen …**	in sehr hohem Maße	in hohem Maße	zum Teil	in geringem Maße	in sehr geringem Maße	gar nicht
BPa91	… ist umsetzbar.	☐	☐	☐	☐	☐	☐
BPa92	… halte ich für eine wirksame Infektionsschutzmaßnahme.	☐	☐	☐	☐	☐	☐
	**Teilung / Kohortierung des Lehrkräftekollegiums …**	in sehr hohem Maße	in hohem Maße	zum Teil	in geringem Maße	in sehr geringem Maße	gar nicht
BPa101	… ist umsetzbar.	☐	☐	☐	☐	☐	☐
BPa102	… halte ich für eine wirksame Infektionsschutzmaßnahme.	☐	☐	☐	☐	☐	☐
	**Wechsel der Räume durch Lehrkräfte, Schüler*innen bleiben in den Klassenräumen …**	in sehr hohem Maße	in hohem Maße	zum Teil	in geringem Maße	in sehr geringem Maße	gar nicht
BPa111	… ist umsetzbar.	☐	☐	☐	☐	☐	☐
BPa112	… halte ich für eine wirksame Infektionsschutzmaßnahme.	☐	☐	☐	☐	☐	☐
	**Verzicht auf Gruppenarbeiten …**	in sehr hohem Maße	in hohem Maße	zum Teil	in geringem Maße	in sehr geringem Maße	gar nicht
BPa121	… ist umsetzbar.	☐	☐	☐	☐	☐	☐
BPa122	… halte ich für eine wirksame Infektionsschutzmaßnahme.	☐	☐	☐	☐	☐	☐

	**Bereitstellen von Möglichkeiten einer hygienischen Handreinigung …**	in sehr hohem Maße	in hohem Maße	zum Teil	in geringem Maße	in sehr geringem Maße	gar nicht
BPa131	… ist umsetzbar.	☐	☐	☐	☐	☐	☐
BPa132	… halte ich für eine wirksame Infektionsschutzmaßnahme.	☐	☐	☐	☐	☐	☐
	**Gewährleistung eines größeren Abstands zwischen den Tischen in Unterrichtsräumen…**	in sehr hohem Maße	in hohem Maße	zum Teil	in geringem Maße	in sehr geringem Maße	gar nicht
BPa141	… ist umsetzbar.	☐	☐	☐	☐	☐	☐
BPa142	… halte ich für eine wirksame Infektionsschutzmaßnahme.	☐	☐	☐	☐	☐	☐
	**Bereitstellung zusätzlicher (Pausen)räume für Lehrkräfte …**	in sehr hohem Maße	in hohem Maße	zum Teil	in geringem Maße	in sehr geringem Maße	gar nicht
BPa151	… ist umsetzbar.	☐	☐	☐	☐	☐	☐
BPa152	… halte ich für eine wirksame Infektionsschutzmaßnahme.	☐	☐	☐	☐	☐	☐
	**Regelmäßige Desinfektion von Handläufen und Türklinken …**	in sehr hohem Maße	in hohem Maße	zum Teil	in geringem Maße	in sehr geringem Maße	gar nicht
BPa161	… ist umsetzbar.	☐	☐	☐	☐	☐	☐
BPa162	… halte ich für eine wirksame Infektionsschutzmaßnahme.	☐	☐	☐	☐	☐	☐

	**Bewegung nur in festgelegten Kleingruppen (im Schulgebäude) …**	in sehr hohem Maße	in hohem Maße	zum Teil	in geringem Maße	in sehr geringem Maße	gar nicht
BPa171	… ist umsetzbar.	☐	☐	☐	☐	☐	☐
BPa172	… halte ich für eine wirksame Infektionsschutzmaßnahme.	☐	☐	☐	☐	☐	☐
	**Einhaltung einer Zuordnung von Zonen für feste Kleingruppen auf dem Schulhof …**	in sehr hohem Maße	in hohem Maße	zum Teil	in geringem Maße	in sehr geringem Maße	gar nicht
BPa181	… ist umsetzbar.	☐	☐	☐	☐	☐	☐
BPa182	… halte ich für eine wirksame Infektionsschutzmaßnahme.	☐	☐	☐	☐	☐	☐
	**Unterbinden der gemeinsamen Nutzung von Sport- und Spielgeräten …**	in sehr hohem Maße	in hohem Maße	zum Teil	in geringem Maße	in sehr geringem Maße	gar nicht
BPa191	… ist umsetzbar.	☐	☐	☐	☐	☐	☐
BPa192	… halte ich für eine wirksame Infektionsschutzmaßnahme.	☐	☐	☐	☐	☐	☐
	**Einhaltung eines Zutrittsverbots für Personen ohne konkreten Schulbezug …**	in sehr hohem Maße	in hohem Maße	zum Teil	in geringem Maße	in sehr geringem Maße	gar nicht
BPa201	… ist umsetzbar.	☐	☐	☐	☐	☐	☐
BPa202	… halte ich für eine wirksame Infektionsschutzmaßnahme.	☐	☐	☐	☐	☐	☐

	**Einhaltung versetzter Pausenzeiten …**	in sehr hohem Maße	in hohem Maße	zum Teil	in geringem Maße	in sehr geringem Maße	gar nicht
BPa211	… ist umsetzbar.	☐	☐	☐	☐	☐	☐
BPa212	… halte ich für eine wirksame Infektionsschutzmaßnahme.	☐	☐	☐	☐	☐	☐
	**Einsatz zusätzlicher Schulbusse …**	in sehr hohem Maße	in hohem Maße	zum Teil	in geringem Maße	in sehr geringem Maße	gar nicht
BPa221	… ist umsetzbar.	☐	☐	☐	☐	☐	☐
BPa222	… halte ich für eine wirksame Infektionsschutzmaßnahme.	☐	☐	☐	☐	☐	☐
	**Einhaltung von Kriterien, die den Schulbesuch verbieten (z. B. Erkältungssymptome) …**	in sehr hohem Maße	in hohem Maße	zum Teil	in geringem Maße	in sehr geringem Maße	gar nicht
BPa231	… ist umsetzbar.	☐	☐	☐	☐	☐	☐
BPa232	… halte ich für eine wirksame Infektionsschutzmaßnahme.	☐	☐	☐	☐	☐	☐
	**Aussetzen von Klassenfahrten, Schulfeiern und ähnlichen Aktivitäten …**	in sehr hohem Maße	in hohem Maße	zum Teil	in geringem Maße	in sehr geringem Maße	gar nicht
BPa241	… ist umsetzbar.	☐	☐	☐	☐	☐	☐
BPa242	… halte ich für eine wirksame Infektionsschutzmaßnahme.	☐	☐	☐	☐	☐	☐

	**Verlegung von Schulaktivitäten (z. B. Sportunterricht) ins Freie …**	in sehr hohem Maße	in hohem Maße	zum Teil	in geringem Maße	in sehr geringem Maße	gar nicht
BPa251	… ist umsetzbar.	☐	☐	☐	☐	☐	☐
BPa252	… halte ich für eine wirksame Infektionsschutzmaßnahme.	☐	☐	☐	☐	☐	☐
	**Räumliche Entzerrung durch Nutzung / Schaffung zusätzlicher Raumkapazitäten …**	in sehr hohem Maße	in hohem Maße	zum Teil	in geringem Maße	in sehr geringem Maße	gar nicht
BPa261	… ist umsetzbar.	☐	☐	☐	☐	☐	☐
BPa262	… halte ich für eine wirksame Infektionsschutzmaßnahme.	☐	☐	☐	☐	☐	☐
	**Benennung von schulinternen Hygienebeauftragten / Corona-Verantwortlichen …**	in sehr hohem Maße	in hohem Maße	zum Teil	in geringem Maße	in sehr geringem Maße	gar nicht
BPa271	… ist umsetzbar.	☐	☐	☐	☐	☐	☐
BPa272	… halte ich für eine wirksame Infektionsschutzmaßnahme.	☐	☐	☐	☐	☐	☐
	**Messung der Körpertemperatur, um Personen mit erhöhter Temperatur vom Schulbetrieb fernzuhalten …**	in sehr hohem Maße	in hohem Maße	zum Teil	in geringem Maße	in sehr geringem Maße	gar nicht
BPa281	… ist umsetzbar.	☐	☐	☐	☐	☐	☐
BPa282	… halte ich für eine wirksame Infektionsschutzmaßnahme.	☐	☐	☐	☐	☐	☐

		in sehr hohem Maße	in hohem Maße	zum Teil	in geringem Maße	in sehr geringem Maße	gar nicht
	**[Freitext] …**<br>Hinweistext: Wenn Sie eine weitere, noch nicht genannte Infektionsschutzmaßnahme bewerten wollen, tragen Sie diese gerne in das Freitextfeld ein.	in sehr hohem Maße	in hohem Maße	zum Teil	in geringem Maße	in sehr geringem Maße	gar nicht
BPa291	… ist umsetzbar.	☐	☐	☐	☐	☐	☐
BPa292	… halte ich für eine wirksame Infektionsschutzmaßnahme.	☐	☐	☐	☐	☐	☐

	**[Freitext] …**<br>Hinweistext: Wenn Sie eine weitere, noch nicht genannte Infektionsschutzmaßnahme bewerten wollen, tragen Sie diese gerne in das Freitextfeld ein.	in sehr hohem Maße	in hohem Maße	zum Teil	in geringem Maße	in sehr geringem Maße	gar nicht
BPa301	… ist umsetzbar.	☐	☐	☐	☐	☐	☐
BPa302	… halte ich für eine wirksame Infektionsschutzmaßnahme.	☐	☐	☐	☐	☐	☐

BPaBP	Was bräuchten Sie, um sich an Ihrem Arbeitsplatz bestmöglich vor einer COVID-19-Infektion geschützt zu fühlen?
	[Freitext]

5b) Beispiele bewährter Maßnahmen zur Umsetzung des Bildungsauftrags

Im Folgenden geht es darum genauer zu erfahren, welche Maßnahmen sich zur Umsetzung des Bildungsauftrags während der COVID-19-Pandemie bewährt haben (denken Sie dabei bitte sowohl an Phasen des Distanzunterrichts als auch an Phasen des Präsenzunterrichts). Ziel ist es, die am besten bewerteten Konzepte systematisch zu erfassen, um daraus Maßnahmenempfehlungen abzuleiten.

5b)							
	**Wie schätzen Sie über unterschiedliche Situationen Ihres Schulalltags hinweg die Umsetzbarkeit sowie die Wirksamkeit folgender Maßnahmen zur Umsetzung des Bildungsauftrags während der COVID-19-Pandemie im Schulbetrieb ein?**						
	**Einheitliches Konzept zur Umsetzung digitalen Unterrichts …**	in sehr hohem Maße	in hohem Maße	zum Teil	in geringem Maße	in sehr geringem Maße	gar nicht
BPb11	… ist umsetzbar.	☐	☐	☐	☐	☐	☐
BPb12	… halte ich für eine wirksame Maßnahme zur Umsetzung des Bildungsauftrags.	☐	☐	☐	☐	☐	☐
	**Bereitstellung technischer Ausstattung …**	in sehr hohem Maße	in hohem Maße	zum Teil	in geringem Maße	in sehr geringem Maße	gar nicht
BPb21	… ist umsetzbar.	☐	☐	☐	☐	☐	☐
BPb22	… halte ich für eine wirksame Maßnahme zur Umsetzung des Bildungsauftrags.	☐	☐	☐	☐	☐	☐

5b)							
	**Weiterbildung zu digitalen Unterrichtsformaten …**	in sehr hohem Maße	in hohem Maße	zum Teil	in geringem Maße	in sehr geringem Maße	gar nicht
BPb31	… ist umsetzbar.	☐	☐	☐	☐	☐	☐
BPb32	… halte ich für eine wirksame Maßnahme zur Umsetzung des Bildungsauftrags.	☐	☐	☐	☐	☐	☐
	**Distanzunterricht per Video-Anruf …**	in sehr hohem Maße	in hohem Maße	zum Teil	in geringem Maße	in sehr geringem Maße	gar nicht
BPb41	… ist umsetzbar.	☐	☐	☐	☐	☐	☐
BPb42	… halte ich für eine wirksame Maßnahme zur Umsetzung des Bildungsauftrags.	☐	☐	☐	☐	☐	☐
	**Rückmeldung zu Lernergebnissen aus digitalen Unterrichtsformaten …**	in sehr hohem Maße	in hohem Maße	zum Teil	in geringem Maße	in sehr geringem Maße	gar nicht
BPb51	… ist umsetzbar.	☐	☐	☐	☐	☐	☐
BPb52	… halte ich für eine wirksame Maßnahme zur Umsetzung des Bildungsauftrags.	☐	☐	☐	☐	☐	☐
	**Regelmäßige Lernerfolgskontrollen im Rahmen digitaler Unterrichtsformate …**	in sehr hohem Maße	in hohem Maße	zum Teil	in geringem Maße	in sehr geringem Maße	gar nicht
BPb61	… ist umsetzbar.	☐	☐	☐	☐	☐	☐

5b)							
BPb62	… halte ich für eine wirksame Maßnahme zur Umsetzung des Bildungsauftrags.	☐	☐	☐	☐	☐	☐
	**Reduktion der Schwierigkeit von Lernerfolgskontrollen …**	in sehr hohem Maße	in hohem Maße	zum Teil	in geringem Maße	in sehr geringem Maße	gar nicht
BPb71	… ist umsetzbar.	☐	☐	☐	☐	☐	☐
BPb72	… halte ich für eine wirksame Maßnahme zur Umsetzung des Bildungsauftrags.	☐	☐	☐	☐	☐	☐
	**Regelmäßiger Austausch mit Schüler*innen …**	in sehr hohem Maße	in hohem Maße	zum Teil	in geringem Maße	in sehr geringem Maße	gar nicht
BPb81	… ist umsetzbar.	☐	☐	☐	☐	☐	☐
BPb82	… halte ich für eine wirksame Maßnahme zur Umsetzung des Bildungsauftrags.	☐	☐	☐	☐	☐	☐
	**Regelmäßiger Austausch mit Erziehungsberechtigten der Schüler*innen …**	in sehr hohem Maße	in hohem Maße	zum Teil	in geringem Maße	in sehr geringem Maße	gar nicht
BPb91	… ist umsetzbar.	☐	☐	☐	☐	☐	☐
BPb92	… halte ich für eine wirksame Maßnahme zur Umsetzung des Bildungsauftrags.	☐	☐	☐	☐	☐	☐
	**Regelmäßiger Austausch innerhalb des Kollegiums …**	in sehr hohem Maße	in hohem Maße	zum Teil	in geringem Maße	in sehr geringem Maße	gar nicht

5b)							
BPb101	… ist umsetzbar.	☐	☐	☐	☐	☐	☐
BPb102	… halte ich für eine wirksame Maßnahme zur Umsetzung des Bildungsauftrags.	☐	☐	☐	☐	☐	☐
	**Einsatz von Tages-, Wochen-, Monatsplänen zur Unterrichtsplanung während des Distanzunterrichts …**	in sehr hohem Maße	in hohem Maße	zum Teil	in geringem Maße	in sehr geringem Maße	gar nicht
BPb111	… ist umsetzbar.	☐	☐	☐	☐	☐	☐
BPb112	… halte ich für eine wirksame Maßnahme zur Umsetzung des Bildungsauftrags.	☐	☐	☐	☐	☐	☐
	**Gewährleistung der Unterrichtsteilhabe aller Schüler*innen …**	in sehr hohem Maße	in hohem Maße	zum Teil	in geringem Maße	in sehr geringem Maße	gar nicht
BPb121	… ist umsetzbar.	☐	☐	☐	☐	☐	☐
BPb122	… halte ich für eine wirksame Maßnahme zur Umsetzung des Bildungsauftrags.	☐	☐	☐	☐	☐	☐
	**Verpflichtender Online-Unterricht …**	in sehr hohem Maße	in hohem Maße	zum Teil	in geringem Maße	in sehr geringem Maße	gar nicht
BPb131	… ist umsetzbar.	☐	☐	☐	☐	☐	☐
BPb132	… halte ich für eine wirksame Maßnahme zur Umsetzung des Bildungsauftrags.	☐	☐	☐	☐	☐	☐

5b)							
	**Verlegung von Schulaktivitäten (z. B. Sportunterricht) ins Freie …**	in sehr hohem Maße	in hohem Maße	zum Teil	in geringem Maße	in sehr geringem Maße	gar nicht
BPb141	… ist umsetzbar.	☐	☐	☐	☐	☐	☐
BPb142	… halte ich für eine wirksame Maßnahme zur Umsetzung des Bildungsauftrags.	☐	☐	☐	☐	☐	☐
	**[Freitext] …**<br>Hinweistext: Wenn Sie eine weitere, noch nicht genannte Maßnahme zur Umsetzung des Bildungsauftrages bewerten wollen, tragen Sie diese gerne in das Freitextfeld ein.	in sehr hohem Maße	in hohem Maße	zum Teil	in geringem Maße	in sehr geringem Maße	gar nicht
BPb151	… ist umsetzbar.	☐	☐	☐	☐	☐	☐
BPb152	… halte ich für eine wirksame Maßnahme zur Umsetzung des Bildungsauftrags.	☐	☐	☐	☐	☐	☐
	**[Freitext] …**<br>Hinweistext: Wenn Sie eine weitere, noch nicht genannte Maßnahme zur Umsetzung des Bildungsauftrages bewerten wollen, tragen Sie diese gerne in das Freitextfeld ein.	in sehr hohem Maße	in hohem Maße	zum Teil	in geringem Maße	in sehr geringem Maße	gar nicht
BPb161	… ist umsetzbar.	☐	☐	☐	☐	☐	☐
BPb162	… halte ich für eine wirksame Maßnahme zur Umsetzung des Bildungsauftrags.	☐	☐	☐	☐	☐	☐

BPbBP	Was bräuchten Sie, um den Bildungsauftrag während der COVID-19-Pandemie bestmöglich umsetzen zu können?
	[Freitext]

Rekrutierungsweg

REK	Durch wen sind Sie auf unsere Online-Befragung aufmerksam geworden? (Mehrfachnennung möglich)	☐ Schulleiter*in	☐ Kolleg*innen	☐ Gewerkschaft
		☐ Online-Nachrichtenportal	☐ Berufsverband	☐ Die für Ihre Schule / Dienststelle zuständige Landesbehörde
		☐ Sonstige: [Freitext]		
